# Dynamics in the resistant and susceptible peanut (*Arachis hypogaea* L*.*) root transcriptome on infection with the *Ralstonia solanacearum*

**DOI:** 10.1186/1471-2164-15-1078

**Published:** 2014-12-07

**Authors:** Yuning Chen, Xiaoping Ren, Xiaojing Zhou, Li Huang, Liying Yan, Yong Lei, Boshou Liao, Jinyong Huang, Shunmou Huang, Wenhui Wei, Huifang Jiang

**Affiliations:** Oil Crop Research Institute, Chinese Academy of Agricultural Sciences/Key Laboratory of Biology and Genetic Improvement of Oil Crops, Ministry of Agriculture, No 2 Xudong Second Road, Wuhan, 430062 P.R. China; School of Life Sciences, Zhengzhou University, Zhengzhou, 450001 P.R. China

**Keywords:** *Arachis hypogaea* L, *Ralstonia solanacearum*, DEGs, RNA-seq

## Abstract

**Background:**

Bacterial wilt caused by *Ralstonia solanacearum* is a serious soil-borne disease of peanut (*Arachis hypogaea* L). The molecular basis of peanut response to *R. solanacearum* remains unknown. To understand the resistance mechanism behind peanut resistance to *R. solanacearum*, we used RNA-Seq to perform global transcriptome profiling on the roots of peanut resistant (R) and susceptible (S) genotypes under *R. solanacearum* infection.

**Results:**

A total of 4.95 x 10^8^ raw sequence reads were generated and subsequently assembled into 271, 790 unigenes with an average length of 890 bp and a N50 of 1, 665 bp. 179, 641 unigenes could be annotated by public protein databases. The pairwise transcriptome comparsions of time course (6, 12, 24, 48 and 72 h post inoculation) were conducted 1) between inoculated and control samples of each genotype, 2) between inoculated samples of R and S genotypes. The linear dynamics of transcriptome profile was observed between adjacent samples for each genotype, two genotypes shared similar transcriptome pattern at early time points with most significant up regulation at 12 hour, and samples from R genotype at 24 h and S genotype at 48 h showed similar transcriptome pattern, significant differences of transcriptional profile were observed in pairwise comparisons between R and S genotypes. KEGG analysis showed that the primary metabolisms were inhibited in both genotypes and stronger inhibition in R genotype post inoculation. The defense related genes (*R* gene, *LRR-RLK*, cell wall genes, etc.) generally showed a genotype-specific down regulation and different expression between both genotypes.

**Conclusion:**

This transcriptome profiling provided the largest data set that explores the dynamic in crosstalk between peanut and *R. solanacearum*. The results suggested that the down-regulation of primary metabolism is contributed to the resistance difference between R and S genotypes. The genotype-specific expression pattern of defense related DEGs also contributed to the resistance difference between R and S genotype. This study will strongly contribute to better understand the molecular interaction between plant and *R. solanacearum*.

**Electronic supplementary material:**

The online version of this article (doi:10.1186/1471-2164-15-1078) contains supplementary material, which is available to authorized users.

## Background

Peanut (*Arachis hypogaea* L.) is an important crop for oil and protein production in the tropical and subtropical regions of the world, Asia is the top region of peanut planting with the growing area of 11 million ha (http://faostat.fao.org/site/339/default.aspx). The bacterial wilt caused by *Ralstonia solanacearum* is a destructive soil borne peanut disease in Asia. It could cause peanut production reduction at least of 10% even a mass extinction. *R. solanacearum* has a wide host range expanding over more than 200 plant species [1]. The process of *R. solanacearum* infecting plant had been well characterized in model crop, briefly, the *R. solanacearum* penetrated into cortical tissue of host roots, colonized and exploded in numbers, caused a sudden deadly wilt of plant [2–4]. The resistant breeding is the most ideal strategy for controlling bacterial wilt with great benefit of economy and environmental protection [1]. However, even in the resistance varieties, *R. solanacearum* can multiply in a high level of number and caused the symptoms of stunted growth, weak wilting and reduced resistance to other pathogens, finally resulted in a potential crop failure [5]. Up to now, the molecular basis of peanut resistance to *R. solanacearum* is poorly understood.

Understanding the complexity of disease resistance will contribute to the development of peanut resistance to bacterial wilt. In the past few decades, the molecular cross-talk between plants and pathogens had been characterized, the intrinsic mechanism of plant resistance to pathogens had been well documented [6–8]. During plant-pathogen interactions, plant evolved a two-tiered innate immunity system to defend against pathogens attack. The host cell surface localized pattern-recognition receptors (PRRs) recognizes pathogen-associated molecular patterns (PAMPs), then activated the PAMP-triggered immunity (PTI) followed by pathogen effector-triggered immunity (ETI). In PTI and ETI, a set of defense response on transcriptome level were activated, and resulted in the arrest of pathogen clone [9–12]. However, the molecular reaction between plant and *R. solanacearum* have received far less attentions.

Efforts had been made in discovering the molecular mechanisms underlying interactions between *Arabidopsis*–, tomato–, potato– *R. solanacearum*, several resistance related genes and enzymes had been well characterized [13–18]. The significant changes on level of transcriptome and proteome were also observed in interaction between plant and *R. solanacearum*[19–24]. Especially, the mechanism of silicon in priming tomato resistance to *R. solanacearum* has been systematically studied [18, 21, 24], roles of cell wall proteins in tomato defend against *R. solanacearum* were well discussed [25–30]. These have provided preliminary understanding of molecular mechanism of plant response to *R. solanacearum.* Up to now, the resistant mechanism of plant to *R. solanacearum* is obscure, the literature and molecular resources available for plant resistance to *R. solanacearum* remain to be enriched. About 68, 094 ESTs differentially expressed in plant after *R. solanacearum* challenge were identified in previous study (http://www.ncbi.nlm.nih.gov/nucest/?term=ralstonia). The present informations are too poor to clearly shed light on the mechanism behind plant resistance to *R. solanacearum*. The genetic control factors that determined the consequence between plant and *R. solanacearum* are not yet fully identified, and changes in the global transcriptome of plant resistance to *R. solanacearum* is yet to be explored. It is still a great challenge to isolate genes by map-based cloning for the huge genome size of peanut. The traditional methods of sequencing cDNA clones resulted in the loss of rare transcript with inefficient cost, low throughput and lack of quantitation of the transcripts. Recent years, RNA sequencing technologies were developed, it is a more comprehensive and efficient way to carry study of transcriptome level on detecting the expression pattern, explore new exons and novel genes [31, 32]. Especially, the application of this technology is not limited to the prior knowledge of genomic sequence, it had been successfully applied in peanut transcriptome study on development and response to stress [33–36].

In the present study, we invested globally and compared the transcriptome profile in the roots of peanut resistant (R) and susceptible (S) genotypes under *R. solanacearum* infection. The dynamic differences of transcriptome profiles in peanut roots under *R. solanacearum* infection were investigated. The specific transcripts related to peanut response to *R. solanacearum* were identified. The possible roles of differentially expressed unigenes (DEGs) were discussed and the resistant mechanism of peanut to *R. solanacearum* was also deduced. In addition, a better understanding of peanut resistance to *R. solanacearum* could be a reference for exploring the resistance to bacterial pathogen in other crop plants. This study also provided a significant transcriptome resource in systemic plant-*R. solanacearum* interactions.

## Results

### Observation of the bacterial number in peanut roots post-inoculation

**Figure 1:**
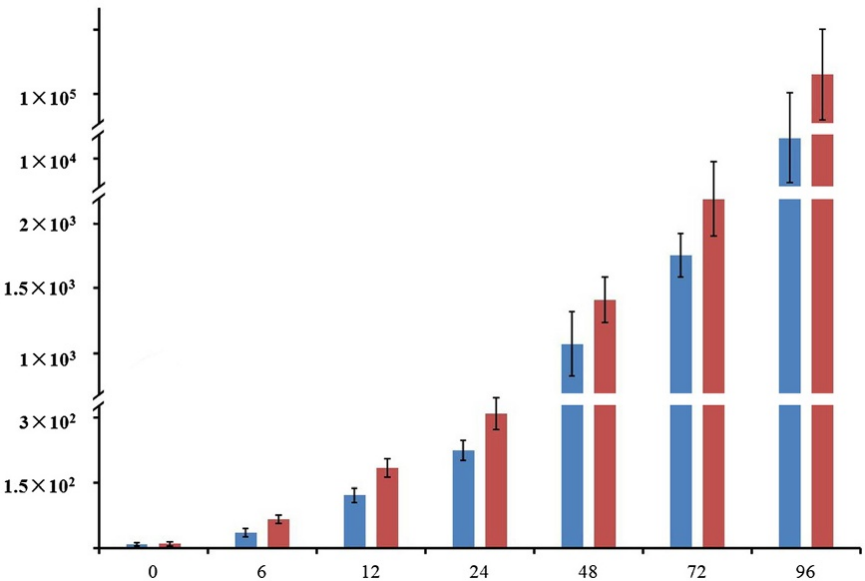
**The population dynamics of** ***Ralstonia solanacearum*** **in peanut roots.** The concentration of *Ralstonia solanacearum* (cfu/g roots) in roots of two genotypes were measured at 0, 6, 12, 24, 48, 72 and 96 hours post inoculation with 10^7^ cfu/ml bacterial suspensions. The blue and red lines represente R and S genotype.

To investigate the process of *R. solanacearum* colonization, the bacterial concentration was measured at 0, 6, 12, 24, 48, 72 and 96 hours post-inoculation for both genotypes. We compared the dynamic change of bacterial population through plate counting. Although the difference were found firstly at 12 hour post-inoculation, compared with the R genotype, the bacterial showed a not significantly rapid reproduction in roots of S genotype until 96 h post inoculation (Figure 1).

### Transcriptome sequencing and *de novo*assembly

In this study, we performed transcriptome analyses of 14 samples to profile peanut response to *R. solanacearum,* it resulted in a total of 9.86 × 10^3^ M bp (Table 1). The comprehensive reads were assembled into transcripts using paired-end reads, it resulted in the 409, 454 comprehensive transcripts. Under the criteria of more than 50-bp overlap and 90% identity, the transcripts were further assembled into 271, 790 unigenes using TGICL. Total of 63, 452 all-unigenes were clustered by at least two unigenes, with a maximum of 27 unigenes per all-unigene, and the rest 212, 569 were corresponding to single unigenes. The total length of all-unigenes was about 241, 893, 100 bp (about 241.8M) covering 8.64% of peanut genome (2, 800 Mb), the size of unigenes ranged from 201 to 15, 900 bp with an average length of 890 bp, the N50 value were 1, 665 bp (Additional file 1: Figure S1). For each sample, it generated a massive number of unigenes ranged from 40, 032 (S6) to 92, 590 (RC2) (Table 1). The sequencing data generated in this study were deposited in NCBI Sequence Read Archieve under the project ID PRJNA252915.

**Table 1:** **The sumuary of transcriptome**

Library	Number of reads	Single length(bp)	Total length(bp)	High quality length(bp)	High quality(%)	Number of unigenes
RC1	34926732	100	6985346400	6029270845	86.31	84749
RC2	33445235	100	6689047000	5795443100	86.64	92590
R6	41434566	100	8286913200	7072570751	85.35	46015
R12	27490083	100	4948214940	4394096605	88.80	63406
R24	39123772	100	7824754400	6696213334	85.58	40355
R48	35984109	100	7196821800	6207801670	86.26	53932
R72	35662641	100	7132528200	6157181972	86.33	62913
Total	248067138	100	49063625940	42352578277	86.47	158595
SC1	33782747	100	6756549400	5869814476	86.88	72377
SC2	38884609	100	7776921800	6574175418	84.53	72821
S6	38626705	100	7725341000	6468359253	83.73	40032
S12	27424672	100	4936440960	4383105373	88.79	79437
S24	38703178	100	7740635600	6567567469	84.85	54464
S48	37229207	100	7445841400	6444140962	86.55	73864
S72	35793115	100	7158623000	6135094229	85.70	68224
Total	250444233	100	49540353160	42442257180	85.86	156911

### Function annotation

To characterize the unigenes, they were searched against Nr protein database using Blastx. For all 271, 790 unigenes, 92, 147 (33.91%) unigenes had no match with Nr record, 179, 643 (66.09%) unigenes matched protein accessions in Nr database, only 100, 336 of 271, 790 unigenes (36.92%) were assigned valuable Nr annotations (Additional file 2: Table S1, Additional file 3: File S1). For sequence similarity analyses by Blast against Nt database, 109, 845 (40.42%) unigenes had matched significant similar sequences (Additional file 2: Table S1, Additional file 4: File S2). The 45.46% sequences have strong homology at the E-value of ≤1.0e^-50^ of top hit in the Nt database. (Additional file 4: File S2). To get a more overall view of present sequences, they were searched against special database of *Arachis* species, *Glycine max* and *Arabidopsis* with a cutoff of E-value of 1.0E^-10^. For the increasing of peanut transcripts from Sanger and RNA-sequencing, a present largest database for *Arachis* species database (peanut DB) were built (http://bioinfolab.muohio.edu/txid3818v1/) [37]. Up to 109, 968 (47.03%) of 271, 790 unigenes matched 27, 694 contigs of *Arachis* species. 47, 201 unigenes matched to 21, 576 sequence from *Glycine max* and 107, 399 unigenes matched to 8, 299 genes from *Arabidopsis*, respectively (Additional file 5: File S3). It also observed that 1, 936 unigenes matched 1, 063 genes of *R. solanacearum* genome (Additional file 6: File S4). This observation proved a reliable representation of the comprehensive transcriptome in which even *R. solanacearum* transcripts were detected.

To further characterized sequence annotation, all unigenes were searched against COG database to analyze phylogenetically widespread domain families. From present unigenes set, 92, 326 of 271, 790 unigenes have significant homologies in COG database (Additional file 2: Table S1, Additional file 7: File S5). A total of 59, 472 unigenes were assigned to the 25 functions categories (Additional file 8: Figure S2). Among this, the top six categories included “Replication, recombination and repair” (13, 226, 14.36%), “translation, ribosomal structure and biogenesis” (13, 194, 14.29%), “Transcription” (12, 944, 14.01%), “posttranslational modification, protein turnover, chaperones” (10, 646, 11.53%) and “Signal transduction mechanisms” (10, 105, 11.96%). 1, 596 (1.72%) of unigenes were grouped into “defense mechanism”.

Following the Nr annotations, all unigenes were mapped into the records of GO database. 111, 663 of them retrieved 333, 350 annotations from 52 sub-categories of three GO categories. “Biological Process” took up the majority of GO annotations (93, 914, 28.17%), followed by “Molecular Function” (200, 315, 60.09%) and Cellular Component (39, 183, 11.75%) (Additional file 2: Table S1, Additional file 9: Figure S3, Additional file 10: File S6). Due to the limited descriptions, a small proportion of unigenes with GO distributions was determined.

**Figure 2:**
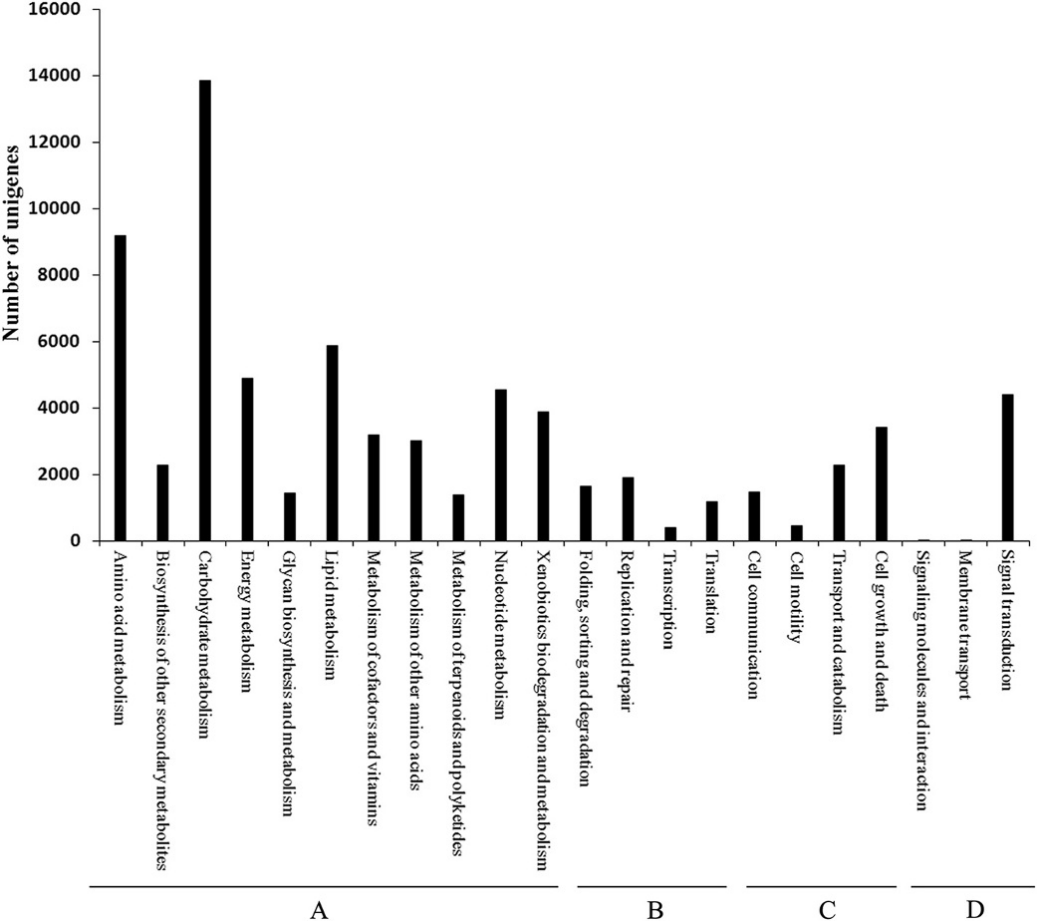
**KEGG analysis of unigeness.** The all-unigenes were assigned into 22 clades of KEGG pathways under five major categories: metabolism **(A)**, Genetic information processing **(B)**, Cellular process **(C)**, Environmental information processing **(D)**.

Finally, the KEGG analyses were performed to identify the biological pathways in peanut root. In total, 70, 762 (26.03%) out of 271, 790 were assigned 237 KEGG pathways (Additional file 11: File S7). These pathways belonged to 22 clades of four categories of “Metabolism” (53, 579/271, 790, 19.71%), “Genetic information processing” (5, 118/271, 790, 1.88%), “Environment information processing” (7, 628/271, 790, 10.63%), “Cellular process” (4, 437/271, 790, 1.63%) (Figure 2). Among them, the top six pathways were, “Carbohydrate Metabolism”, “Amino Acid Metabolism”, “Lipid metabolism”, “Energy metabolism”, “Nucleotide metabolism” and “Signal Transduction”.

### Cluster analysis of transcriptome

**Figure 3:**
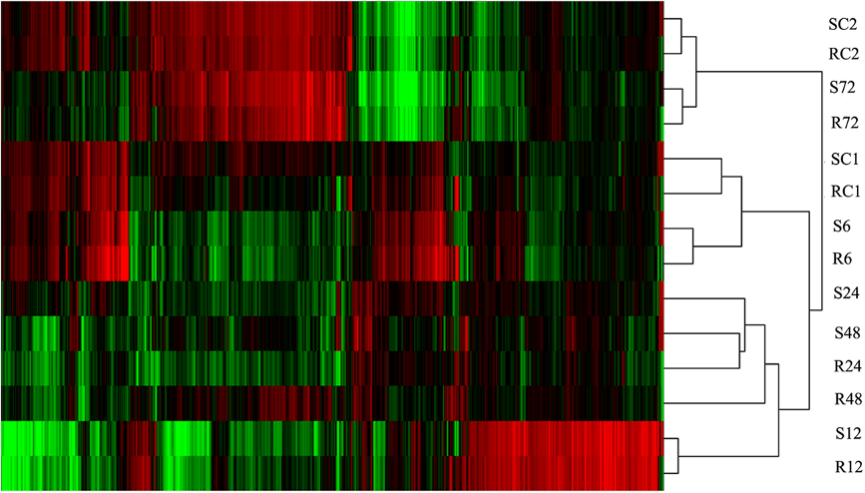
**Transcriptome profile analysis of both control and inoculated samples with** ***R. solanacearum*** **.** Clustering algorithms and Treeview analysis were done for 14 sub-libraries. For both control and inoculated samples, similar gene expression profiles were shared by samples from paired time points. Green indicates the low expression, black indicates intermediate expression, and red indicates the high expression.

To get a global view of transcriptional profiles of peanut response to *R. solanacearum* colonization, Clustering algorithms and Treeview were used to analyze the transcripts profiles for the 14 samples (Figure 3). They showed similar profiles at paired time points between both genotypes and distinct sample-specific profiles in each genotype. Generally, the similar transcriptome patterns were found among control samples (RC1 Vs SC1, RC2 Vs SC2) and earlier inoculated samples (R6 Vs S6, R12 Vs S12), respectively. The up-regulated genes appeared a peak at 12 hour post-inoculation. Interestingly, the samples of R24 and S48 shared similar transcriptome pattern.

### Identification of DEGs

**Figure 4:**
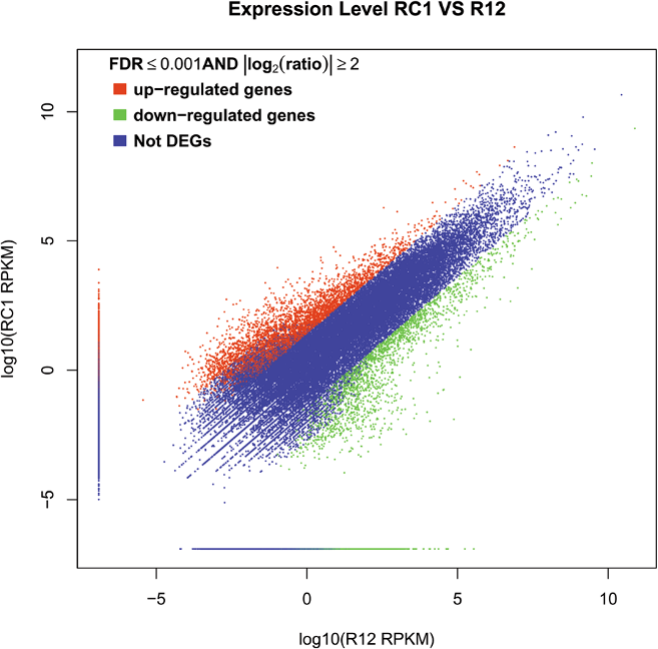
**The scatter plot comparing the gene expression levels between RC1 and R12.**

In present study, after the multiple comparisons, the DEGs were identified under the two criteria, the average fold change was at less equal to or more than two with the P-value under 0.01 and FDR ≤ 0.001 (For example of R12 Vs RC1, Figure 4). The differential comparisons between control and inoculated samples resulted in the RD and SD series data sets (Additional file 12: Table S2, Additional file 13: File S8). These ten series data sets represented the DEGs responding to *R. solanacearum* challenge of both genotypes. The comparison between inoculated samples resulted in D series data sets, which represented the DEGs between S and R genotype in response to *R. solanacearum* infection (Additional file 12: Table S2).

**Figure 5:**
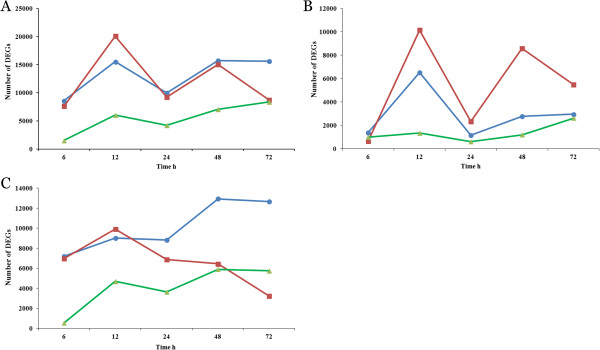
**The dynamic changes of number of DEGs at series time points.** The dynamic trends of DEGs number for total, up-regulated and down-regulated DEGs were showed in Figure **A**, **B** and **C**, respectively. The red lines represent the S data sets, and the blue lines represent the R data sets, the green lines represent the D data sets.

The dynamic trends of DEGs in RD, SD and D data sets were investigated. It was obviously observed that remarkable changes of transcriptome profile occured at 12 hour (Additional file 14: Table S3, Figure 5A). The number of down-regulated DEGs was markedly more than those up-regulated at first three time points in S and all five time points in R genotype (Additional file 14: Table S3). The total DEGs and up-regulated DEGs showed similar changing trends through 6, 12, 24 and 48 h (Figure 5A, B). The distinct dynamic trends were observed for the down-regulated DEGs. For D series data sets, the numbers of DEGs decreased sharply and persistently went up through all time points, indicating that considerable parts of DEGs were shared by two genotypes and more and more evident changes in transcriptome profiles during the process of *R. solanacearum* multiplication between R and S genotype (Figure 5C).

**Figure 6:**
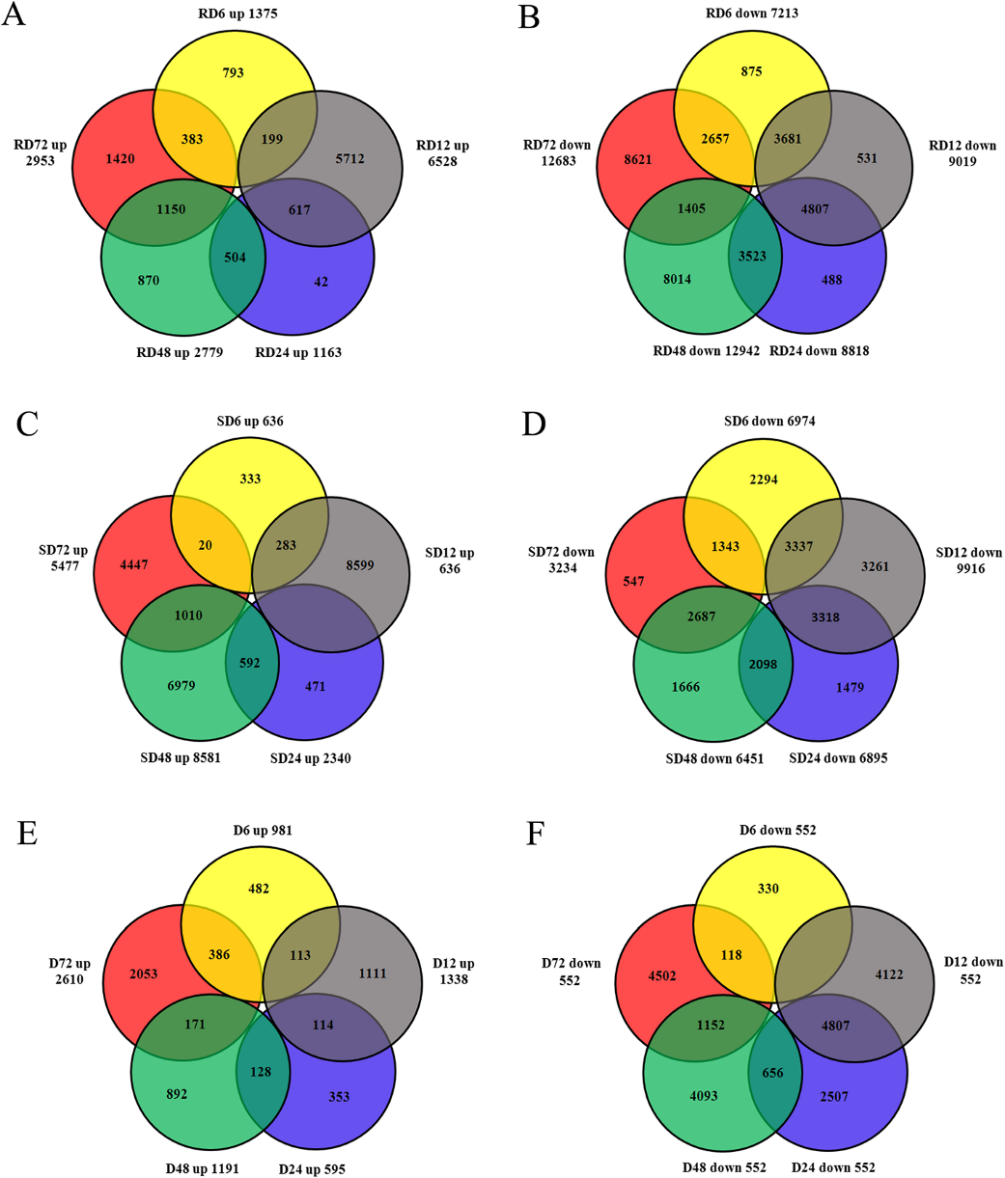
**The distribution of up-and down-regulated DEGs between neighboring data sets.** The numbers of DEGs that were exclusively up- or down-regulated in each data sets were shown in circle. The numbers of DEGs shared by neighbor data sets were shown in the overlapping regions. The total numbers of up- or down-regulated genes at each time point were shown outside the circles. **A** and **B** represent DEGs up-regulated, **C** and **D** represent DEGs down-regulated in R and S genotype post inoculation; **E** and **F** represent DEGs up- and down-regulated in R genotype than that in S genotype.

The property of up- and down-regulated DEGs between neighboring data sets was also investigated. The percentage of DEGs exclusive to their own data sets was showed (Figure 6). For up-regulated DEGs, they were more exclusive to data sets of 6 and 12 in RD and 6, 12, 48 and 72 in SD and less overlap in their neighboring data sets (Figure 6A, B). For down-regulated DEGs, they showed more community in neighboring data sets of 6, 12 and 24 h in RD and 6, 12, 24 and 48 h in SD (Figure 6C, D). The data sets of RD24 and SD24 owned the least exclusivity DEGs, they were mostly shared by data sets of 12 and 48 h (Figure 6E, F).

### The Co-expression pattern of DEGs

To identify genes shared same expression pattern, the co-expression patterns of DEGs were further investigated in RD and SD data sets (Additional file 15: Table S4) and D data sets (Additional file 16: Table S5). It uncovered various and genotype-specific expression patterns of DEGs, and the down-regulation and less share of co-expressed DEGs were dominated in both genotypes. The most popular expression patterns shared by both genotypes were down-regulated meanwhile at 6, 12 and 24 h, there were 1, 979 and 1, 911 DEGs in R and S genotype respectively, 852 DEGs were shared by both genotypes.

### Experimental verification of DEGs

To verify the reliability of sequencing-based approach in identifying *R. solanacearum* -responsive genes, we monitored the expression pattern of twelve candidate DEGs at the five time points post inoculation for both genotypes using qRT-PCR. These candidate DEGs included genes that were proved to be related to defend response in other plant species, such as *R* genes and genes involving in ethylene signal transduction pathways. Their expression showed basically linear correlation to the results of the RNA- sequencing (Additional file 17: Table S6, Additional file 18: Figure S4).

### The annotation of DEGs

**Figure 7:**
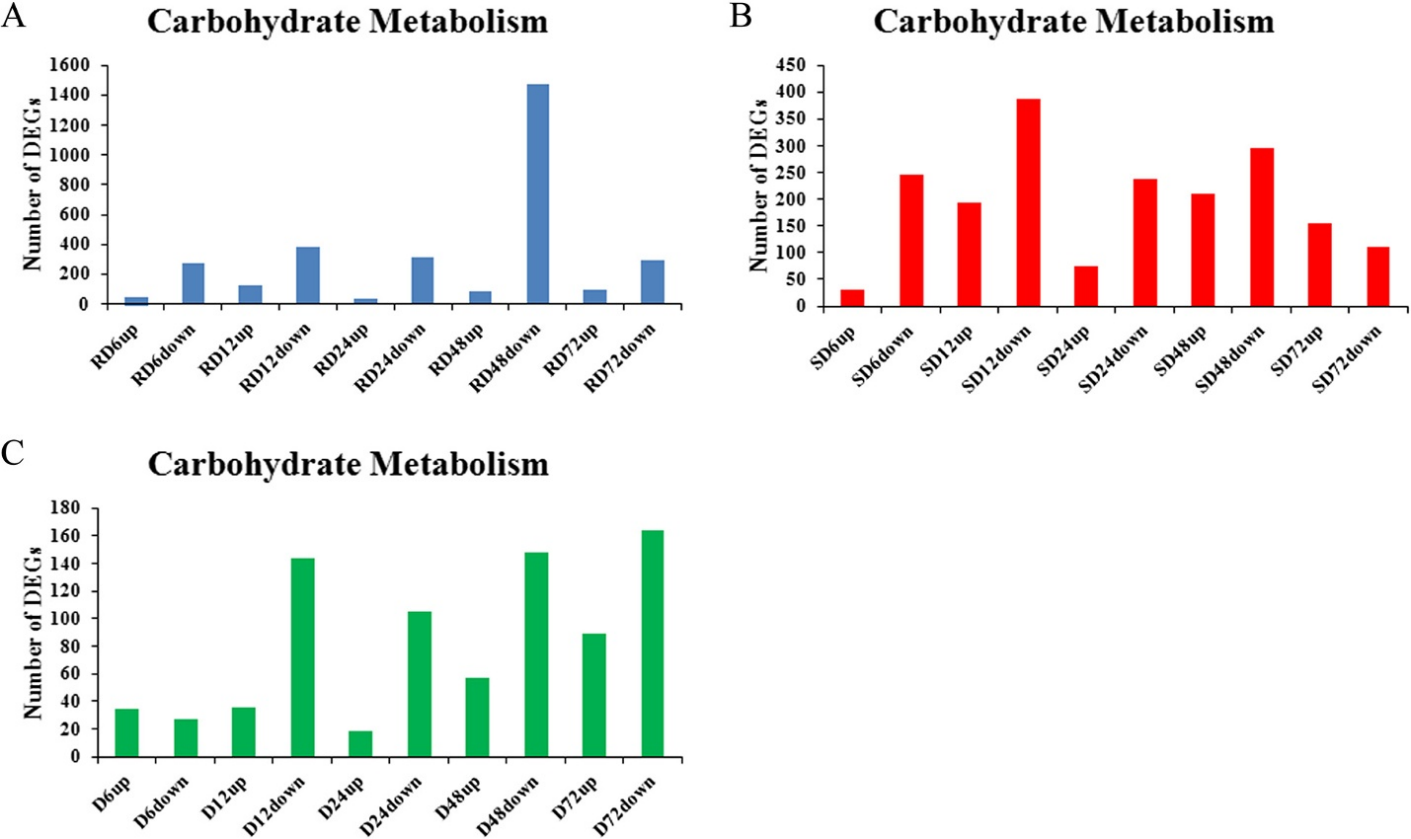
**KEGG enrichment analyses of Carbohydrate metabolism.** The enrichment of DEGs in pathways of Carbohydrate metabolism in R data sets **(A)**, S data sets **(B)** and D data sets **(C)**.

For functional annotation of DEGs, the KEGG enrichment analyses were mainly referenced. The enrichment analyses showed that more DEGs with down-regulation were involved in “Carbohydrate metabolism” than those with up-regulation at 6, 12, 24 and 48 h in S genotype and all five time points in R genotype, with significant enrichment at 48 h in R and 12 h in S genotype, respectively (Figure 7A, B). For D series data sets, the more DEGs with down-regulation were involved in “Carbohydrate metabolism” at 12, 24, 48 and 72 h (Figure 7D). The enrichment analyses indicated that the “Carbohydrate metabolism” was inhibited post inoculation in both genotypes and more extensive inhibition in R genotype. The main concerned pathways were “Glycolysis/Gluconeogenesis”, “Citrate cycle”, “Starch and sucrose metabolism, “Propanoate metabolism” and “Pyruvate metabolism”. DEGs involved in Carbohydrate metabolism were vastly inhibited in both genotypes at more than three time points with more than 100 fold of expression decrease (the Log_2_ ration is 8–11), including genes which encoded pyruvate dehydrogenase E1 component (Ahy247968 and Ahy147270), isocitrate dehydrogese (Ahy068189, Ahy142215 and Ahy224761), malic enzyme (Ahy025916 and Ahy117193), fructosidase (Ahy098542) and phosphoglucose isomerase (Ahy023653). Remarkably, the most dominant expression pattern were observed on the DEGs having domain of sugar transporter (IPR005829), almost all DEGs (22 of 26) encoding sugar transporter were down-regulated with same expression pattern in both genotypes. These genes take fundamental roles in Carbohydrate metabolism (Additional file 19: File S9).

Similar results were observed in metabolism of energy and lipid, DEGs with down-regulation were more than those with up-regulation at 6, 12 and 24 h in S genotype and all time points in R genotype (Additional file 20: Figure S5 and Additional file 21: Figure S6). For D series data sets, the more DEGs with down-regulation involved these two pathways than those with up-regulation at 12, 24 and 48 h. The main related pathway was “Oxidative phosphorylation” and “Fatty acid metabolism”, the expressions of DEGs these two pathways were decreased hundreds of times than those in S genotype (Additional file 19: File S9).

Interestingly, it observed that the numbers of the up-regulated DEGs were close to or more than those of down-regulated DEGs at 12 and 48 h in S genotype. However, the DEGs with up regulation involved in enrichment analysis were significantly less than those with down regulation, the number declined disproportionately. For example of the DEGs involved in metabolism of carbohydrate, energy and lipid at 12 h, the percentages of up-regulation DEGs were almost half or less than those of down-regulated DEGs, they were 1.90% (193/10, 159) and 3.91% (388/9, 916) for carbohydrate metabolism, 1.75% (178/10, 159) and 3.0% (300/9, 916), 1.22% (124/10, 159) and 2.68% (266/9, 916). Further analysis showed that, compared with down-regulated DEGs, low percentage of KEGG annotations occured on up-regulated DEGs in R genotype at 12 h and in S genotype at 12 and 48 h.

In D data set, the DEGs showed always down or up regulation through all five time points with IPR annotation were list here (Tables 2 and 3). Due to the lack of informative annotations, only 12 of 57 with up-regulation and 14 of 47 with down-regulation had IPR annotation, one *R* gene (Ahy163031) showed higher expression in R than in S genotype through all five time points. DEGs encoding leucine-rich repeat (Ahy086504) was significantly down-regulated in S genotype, two member of myb family (Ahy142850, Ahy002012) and zinc finger (Ahy005578, Ahy003448) were significantly down- and up-regulated in S than in R genotype.

**Table 2:** **The DEGs with always up-regulation in D data set**

Gene ID	Fold change					Annotation
	D6	D12	D24	D48	D72	
Ahy157678	8.53	7.52	9.05	14.88	7.59	Zinc finger, BED-type predicted
Ahy062178	3.95	13.13	3.78	4.40	4.09	D-binding pseudobarrel domain
Ahy070340	4.37	10.17	4.38	9.24	3.22	Peptidase aspartic
Ahy132030	3.66	4.50	3.38	2.74	3.42	Bestrophin/UPF0187
Ahy140110	2.05	2.15	2.55	1.36	1.47	Domain of unknown function
Ahy179541	7.09	13.75	7.30	12.82	13.41	Domain of unknown function
Ahy157778	1.84	1.65	1.08	1.18	1.22	Proteise inhibitor I25,
Ahy173735	1.63	1.50	1.53	1.89	1.09	LURP1-like
Ahy124304	2.66	2.61	3.10	2.47	2.60	DP-dependent oxidoreductase
Ahy114746	3.03	11.09	3.16	3.60	3.31	Enoyl-CoA hydratase/isomerase
Ahy142850	11.70	11.10	12.17	12.34	-	Myb transcription factor
Ahy005578	3.20	2.63	4.51	2.87	2.64	Zinc finger

**Table 3:** **The DEGs with always down-regulation in D data set**

Gene ID	Fold change					Annotation
	D6	D12	D24	D48	D72	
Ahy019568	3.24	2.47	3.67	2.90	3.81	Ubiquitin thiolesterase 1-like
Ahy135016	1.94	1.02	1.13	1.24	1.06	ATPase-like
Ahy170931	2.87	4.60	1.20	2.17	4.44	FAR1 D binding domain
Ahy003109	5.60	3.39	2.95	2.41	3.54	Sugar nucleotide epimerase
Ahy149227	13.2	12.3	10.2	10.1	9.74	Domain of unknown function
Ahy014553	8.31	7.39	5.65	5.58	6.34	Protein kinase
Ahy146493	2.87	2.43	2.60	3.71	2.00	Transferase
Ahy163031	2.50	2.84	2.20	1.02	2.54	Disease resistance protein
Ahy000245	1.46	2.30	1.71	1.79	2.78	AAA + ATPase
Ahy002012	6.23	9.89	10.6	11.3	11.1	Myb transcription factor
Ahy003448	8.35	5.86	5.65	9.22	9.01	Zinc finger
Ahy123022	2.22	2.26	1.41	2.37	9.32	Ribosomal protein L7
Ahy134649	11.2	12.0	5.48	10.5	4.76	11-S seed storage protein
Ahy086504	11.4	11.6	11.5	11.7	12.3	Leucine-rich repeat

### The expression patterns of defense-related genes

The expression patterns of genes taking key roles in plant resistance to pathogens were investigated, including those of the NBS-LRR (*R*) genes, the genes encoding leucine-rich repeat receptor-like protein (LRR-RLK), MAP kinase and WRKY factors, the DEGs related to ADP ribosylation, the genes involved in synthesis of phytoalexins including lectin, terpenoid and chalcone, and the genes involved in ethylene, salicylic acid and cytokinkins signaling pathways, and the factors involved in defense pathways such as reactive oxygen species (ROS), cell wall proteins and pathogenesis-related proteins (PR proteins).

**Figure 8:**
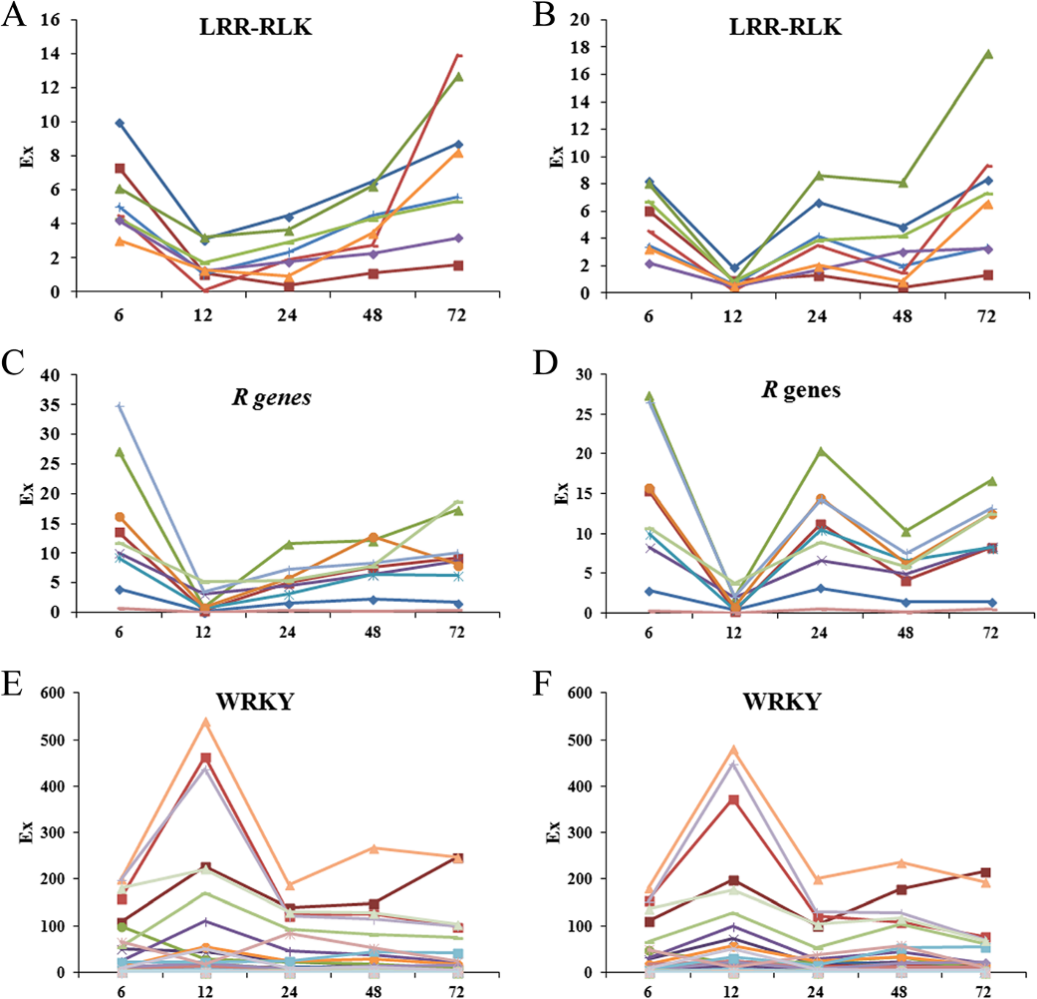
**The expression pattern of DEGs encoding LRR-RLK,** ***R genes*** **and WRKY.** The DEGs encoding LRR-RLK and R genes showed genotype-specific expression pattern. The DEGs encoding WRKY showed similar expression pattern in both genotypes. **A**, **C** and **E** indicate R genotype; and **B**, **D** and **F** indicate S genotype.

Total 64 DEGs with domain of LRR-RLK (IPR001611) were identified (Additional file 22: File S10). Almost all of them were down-regulated in both genotypes, only two of them were up-regulated at 12 or 48 h in both genotypes. Both genotypes showed distinct expression patterns with often down regulation at 12, 24 and 48 in R and 12 and 48 h in S genotype (Figure 8A, B). Compared with S genotype, nineteen were up-regulated at 6 and 48 h in R genotype. Especially, one DEG (Ahy086504) was significant up-regulated at all five time points, without detection in all samples including control samples of S genotype. Six DEGs encoding FLS2 was a documented well LRR-RLK.

Total 168 NBS-LRR type *R* genes were identified (Additional file 22: File S10). They were mainly down-regulated continuously at 12, 24 and 48 in R genotype and intermittently down-regulated at 12 and 48 h in S genotype (Figure 8C, D), and showed slightly up- or down-regulation between R and S genotype at single time points. Compared to S genotype, only one (Ahy163031) was up-regulated at five time points in R genotype, the DEGs encoding *Glycine max R* gene *RPM1* (Ahy090925) and one *R* gene (Ahy077818) were significantly up-regulated (above 8 fold change) at 12 h in R genotype. Interestingly, total 35 DEGs encoding *Medicago truncatula* disease resistance protein RPS1, twenty-eight of them were shared by both genotypes.

Total 30 DEGs encoding WRKY factor (IPR003657) were identified (Additional file 22: File S10). Two DEGs, Ahy114509 and Ahy109642 were down-regulated at all five time points in R genotype and without expression difference in S genotype, 11 DEGs were meanwhile up-regulated at 12 h in both genotypes including DEGs encoding WRKY3, WRKY4, WRKY33, WRKY40, WRKY42, WRKY65 and WRKY70 of *Glycine max*. Four DEGs were significantly down-regulated, WRKY50 (Ahy108250) at 12 h and WRKY20 (Ahy066190) at 48 h in R genotype, WRKY17 (Ahy012174) at 12 h and WRKY48 (Ahy099173) at 48 h in S genotype. Compared with the expression pattern of *R* genes, the expression of *WRKY* showed an opposite pattern with up-regulation at 12 h when the *R* genes were down-regulated (Figure 8E,F).

Total 22 DEGs encoding MAP kinase (IPR003527) were identified, most of them were down-regulated at 12 and 24 h in R genotype and 6, 12, 24 and 48 h in S genotype (Additional file 22: File S10). DEGs encoding MEK2 were down-regulated in both genotype, DEGs encoding MEK4 was up-regulated at 6 h in R genotype. Similar expression pattern were observed in calcium-dependent protein kinases (IPR020636), they were mainly down-regulated in both genotypes (Additional file 22: File S10).

Five DEGs encoding ADP-ribosylation factor (IPR005502) were identified with down regulation in R or S genotype (Additional file 22: File S10). And one (Ahy245540) was up-regulated post inoculation and with no detection in control samples in both genotypes. Compared with S genotype, the expression of Ahy245540 was down-regulated at 48 h and then significantly up-regulated at 72 h in R genotype.

For the DEGs involving in the biosynthesis of phytoalexins, most of them were down-regulated at both genotypes with different expression patterns at later stages. For DEGs encoding domain of lectin (235 DEGs), compared with control, the down-regulation is mainstream in both genotypes (Additional file 23: File S11). Interestingly, all eleven DEGs with ricin B lectin domain were down-regulated in R genotype meanwhile five of them were significantly up-regulated in S genotype.

Twenty-one DEGs encoding chalcone synthase were significant down-regulated at 6 h in R genotype and at 12 h in S genotype (Additional file 23: File S11). For DEGs encoding terpenoid synthase, significant down-regulated occured 12 h in both genotypes (Additional file 23: File S11).

In ROS, the DEGs encoding peroxidase (POD) were down regulated with distinct expression pattern in both genotypes and showed significant up-regulation in S genotype when compared to R genotype (Additional file 23: File S11). The expression pattern of cell wall protein also showed differential regulation (Additional file 22: File S11). DEGs encoding callose synthase (CalS) were down regulated in both genotypes and with higher expression in S genotype when compared to R genotype. On the contrary, DEGs encoding pectinesterase (PE) and Polygalacturonase (PG) were down-regulated in S genotype, one *PG* (Ahy107854) were significant down-regulated at 48 h in S genotype when compared to R genotype, both are key enzymes in cell wall degradation (Additional file 23: File S11). Interestingly, in the COG annotation, the DEGs involved in cell wall biosynthesis generally showed significant up regulation in S than in R genotype (Additional file 24: Figure S7). DEGs encoding PR protein showed different regulation. Compared to R genotype, DEGs encoding *PR-2* (β-1,3-glucanase, Ahy269290) were no detected in all samples (except SC1) in R genotypes with a significant up-regulation at 72 h in S genotype, *PR-1* (Ahy193681) and *PR-5* (Thaumatin-like protein, Ahy119583) were significantly down-regulated at 48 and 72 h respectively in S genotype, one *PR-10* (Ahy120907) was up-regulated at 6 and 48 h respectively in S genotype, one *PR-10* (Ahy249270) was significantly down regulated in both genotypes with no difference between them.

### Expression patterns of the genes in plant hormone signaling pathway

Total 67 ethylene responsive factors (IPR001471) were identified and showed distinct expression pattern (Additional file 25: File S12). DEGs encoding ERF4 and ERF6 were up-regulated in both genotypes. Three DEGs encoding salicylic acid carboxyl methyltransferase (SAMT), all were down-regulated at R and two were down-regulated in S genotype (Additional file 25: File S12).

The distinct expression patterns of DEGs involving in plant hormone occured on DEGs involved in biosynthesis of zeatin. Compared with control, most DEGs encoding zeatin showed up-regulation but no expression changes at 6 h in both genotypes (Additional file 25: File S12). There were 10 DEGs involving in zeatin biosynthesis in both genotypes and another four were detected only in S genotype, and only one was down-regulated in both genotypes and two were down-regulated in S genotype.

## Discussion

### Assembly and annotation of Transcriptome

RNA-seq technologies are powerful for de novo transcripts assembly, identification of novel genes and analysis of transcriptional profiles [38, 39]. In present study, RNA-seq enabled the generation of huge amounts of sequence reads, for characterization of the transcriptional changes of peanut roots under *R. solanacearum* challenges. More than 90 Gbp were generated. The parameters of present transcriptome were far more than those of previous reports in the number of total bp and reads, value of N50, average length of unigenes and coverage of genome sequence. The characters of other transcriptome researches were shown here: covering 211 Mbp of both genomes and 21, 714 contigs with average length of 494 bp for *Arachis* wild species [35], 27.2 million paired-end reads and 59, 077 unigenes with average length of 619 bp and N50 value of 823 [34], 114.87 Mbp of genome sequence, 74, 974 contigs with average length of 859 bp and N50 value of 974 [36], and the coverage of genome sequence were also more than the data from published database which represented 34.41 Mb (http://www.ncbi.nlm.nih.gov/UniGene/UGOrg.cgi?TAXID=3818)) and 33.97 Mb (http://bioinfolab.muohio.edu/txid3818v1/) genome sequences, respectively. It is well known that due to the lack of available annotations for the limited basic research in peanut, the huge number of unigenes (more than 33.91% of total unigenes) had no any matches in Nr database, it added the difficulty of evaluation and understanding of peanut transcriptome.

### Similar transcriptome profiles between R and S genotypes

We performed a time course transcriptome analysis to investigate the roots global response between peanut R and S genotypes during *R. solanacearum* colonization. The previous reported that the plant resistance to *R. solanacearum* were mainly focused on stem (18,24,29) as well as our studies (5), however, the inhibition of root development was also observed in apparently healthy peanut (5), our primary study had revealed a significant difference on root bacterial population at 96 h post inoculation between R and S genotype (Figure 1). So study on the roots response to *R. solanacearum* could contribute to comprehensively understand the mechanism of plant resistance to *R. solanacearum.* The cluster analyses suggested a high synchronization in root development of both genotypes (RC1 Vs SC1, RC2 Vs SC2) and early response to *R. solanacearum* infection (R6 Vs S6, R12 Vs S12). Unexpectedly, the cluster analysis failed to build similarity between the transcriptome profiles of RC1 Vs RC2 and SC1 Vs SC2, showed a dramatic transcriptome change even in the developmental stage of root during only in 72 hours. Interestingly, the similarity patterns of transcriptomes were shared by R24 and S48, which indicated an earlier and more rapid molecular response to *R. solanacearum* infection in R genotype. Although the great difference of an order of magnitude of the bacterial population was observed at 96 hour post inoculation, however, the different transcriptomes were observed as early as 24 h post inoculation, it could be deduced that the resistance to *R. solanacearum* was determined earlier in R genotype before the multiplication of bacteria was inhibited in roots.

### The primary metabolisms were inhibited after inoculation in both genotypes

For RD, SD and D series datasets, the enrichment of DEGs acted in primary metabolism was observed by KEGG analyses. The collective behavior of DEGs indicated that the primary metabolisms were inhibited at early stage in S and whole stage in R genotype, and more evident inhibition of primary metabolism occured on R genotype. Up to now, the roles of primary metabolism in plant defense are not clear and less characterized [40, 41]. Although a number of studies showed that the genes acted in primary metabolism were up-regulated and identified as positive roles in plant resistance response [41]. Interestingly, in the cases of wheat response to powdery mildew and *Arabidopsis thaliana* response to *Pseudomonas syringae* pv. *Tomato*, the genes acting in primary metabolism (glycolysis, the Krebs cycle and the pentose phosphate pathway) were down-regulated, the switchs from primary metabolism to defense metabolism were observed [42, 43]. The change of primary metabolism in host plant caused by pathogen had been documented [40, 44, 45]. It is a consensus that the host plant defense response to pathogen and inhibition growth of pathogen is cost-intensive, the nutrition and energy were transported from primary metabolism to the defense interaction [41]. However, it also showed a very complicated regulation of genes involving in the primary metabolism for their up- or down-regulation even in the same pathways [44, 45]. Interestingly, in present study, the expression pattern of DEGs encoding sugar transporter (IPR005828 ) were opposite to those were previous reported. The sugar transporter were induced to supply nutrient for the pathogen colonization in the cases of *Arabidopsis* to bacterium *Pseudomonas syringae* pv. *tomato* DC3000 and powdery mildew fungus *G. cichoracearum*, rice to *Xanthomonas oryzae* pv. *Oryzae*[46, 47]. It seemed that the peanut reduced the nutrition supply to *R. solanacearum* by down-regulation of sugar transport. In the case of peanut bacterial wilt*,* even in the incompatible interactions between peanut R genotype and *R. solanacearum*, the concentration of *R. solanacearum* is up to the 10^8^cfu/ml even in the R genotype during a long period post inoculation. It could be assumed that the more nutrition and energy was switched to the defense pathways by more intensive and longer-lasting down-regulation of primary metabolism in R genotype, and the down-regulation of primary metabolism is contributed to the resistance difference between R and S genotypes.

### The PTI and ETI were partly suppressed in both genotypes

The molecular event occured in ETI and PTI had been documented and the key components of PTI and ETI were characterized well [48–50]. The sequential molecular events caused by pathogens were perception of PAMP by PRR, and then activation of the MAPK cascade followed the activation of WRKY-type transcription and *R* gene. And the leucine-rich repeat receptor kinases (LRR-RK) were identified as one of PRR. In *Arabidopsis*, the LRR-RLK, named FLS2, can bind bacterial flagellin peptides and contribute to resistance [51]. FLS2 is a component of the preexisting recognition system, its expression was not affected by flagellin. However, in present study, the expressions of DEGs encoding LRR-RLK including FLS2-like kinase were mainly down-regulated, and one LRR-RLK (Ahy086504) was not detected in all samples from S genotype, the role of Ahy086504 deserves to be further studied. After perception of PAMP, the MAPK cascade was activated and MKK4 acted positively and MKK2 acted negatively in PTI in *Arabidopsis*[52, 53]. The expression pattern indicated that a similar regulation of MAPK component occured on the interaction of peanut to *R. solanacearum* like that in *Arabidopsis* to bacterial pathogen. The MAPK cascade then activated WRKY-type transcription factors which act in a complex defense response network as both positive and negative regulators in plant immunity [54]. The WRKY27 took negative role in *Arabidopsis* response to *R. solanacearum*, on the contrary, the CaWRKY27 promoted tobacco resistance to *R. solanacearum*[14, 55]*.* The CaWRKY40 took a positive role in pepper resistance to *R. solanacearum*[56]*.* The WRKY70 were positively regulated in *Brachypodium distachyon* response to pathogens [57]. In this study, the identified DEGS encoding *WRKY* showed distinct expression pattern in both genotypes, WRKY33, -40, -70 were up-regulated and WRKY17, -20, -48 and -50 were down-regulated, it could be deduced that the *WRKY*s acted positively and negatively in peanut resistance to *R. solanacearum*, the exact roles of WRKY still need further study. Based on the expression pattern analysis of components in PTI, with consideration of the increased bacterial populations in both genotypes (Figure 1), it seemed that the components of PTI were activated, however, the *R. solanacearum* partly inhibited PTI in both genotypes.

In the followed ETI, the two types of NBS-LRR genes, CC-NBS-LRR and TIR-NBS-LRR were identified and with general down-regulation post-inoculation, and three *R* genes showed higher expression in R than in S genotype, it argued that the NBS-LRR type of *R* genes were responsible for peanut resistance to *R. solanacearum*. The *R* gene was the central component in ETI by detecting and binding to bacterial effectors and triggered the subsequent defense response. More interestingly, one CC-NBS-LRR type of *R* gene, the *RPS1* of *Medicago truncatula* was represented by 35 unigenes with down-regulation in both genotypes. The soybean *RPS1* (CC-NBS-LRR) was identified as the resistance gene to *Phytophthora sojae* which cause root and stem disease in soybean [58]. It raised the suspicion that the *RPS1* mediated partially the resistance of peanut to *R. solanacearum*. The DEGs encoding *Glycine max R* gene *RPm1* (Ahy090925) were also identified with down-regulation in both genotypes and higher regulation in R genotype. The *Arabidopsis RPm1* is a resistance gene responding to *Pseudomonas syringae* pv. *tomato* DC3000, it associates physically with bacterial FLS2 which destabilizes host ADP ribosylation factor AtMIN7 which is required for both PTI and ETI in *Arabidopsis*[59]. The expression pattern of Ahy245540 encoding ADP ribosylation factor indicated that the degradation of ADP-ribosylation was more effectively blocked in R genotype, Ahy090925 and Ahy245540 maybe act like *RPM1* and *AtMIN7* respectively in *Arabidopsis* response to *Pseudomonas syringae* pv. *tomato* DC3000, thus the PTI in R genotype was rescued by ETI.

In PTI and ETI, the salicylic acid (SA) and ET signaling pathway involves in the activation of defense-related genes that contribute to resistance to pathogen [50, 60]. ERFs involved in SA and ET signaling pathways and take positive or negative roles in plant immunity response [60, 61]. In present study, many types of ERF were identified, *ERF1*, *4* and *6* were up-regulated and *ERF3* was down-regulated in both genotypes, this reproduced the results of our previous report, and confirmed the biosynthesis of ET may contribute to peanut resistance to *R. solanacearum*[24]. The plant cytokinins involved in the resistance to pathogen and promoted the accumulation of salicylic acid in plant defense response [62]. In *Arabidopsis*, zeatin involved in the defense response of *R* gene *RPM1* and caused the accumulation of salicylic acid [62, 63]. Recent research showed that SA positively regulated *Arabidopsis* reistance to *R. solanacearum*[64]. Given the up-regulation of DEGs involved in zeatin biosynthesis and down-regulation of *SAMT,* which balances the level of salicylic acid by methylating SA [65], it appeared the promotion of zeatin lead to the accumulation of salicylic acid, both cytokinins and SA synergistically acted in peanut resistance to *R. solanacearum* in both genotypes, but did not contribute to the resistance difference between R and S genotype.

As the result of *R* gene-mediated resistance, the ROS and cell wall proteins as well as PR proteins contributed to a final immune response to pathogen [50, 60]. In present study, *POD* were up-regulated in S than in R genotype, which involved in production of ROS and reinforcement of the cell wall, increased tomato resistance to *R. solanacearum*[18, 24, 29]. Interestingly, compared to R genotype, DEGs involved in cell wall biosynthesis, such as *CalS*, *POD* and DEGs with COG’s annotation of cell wall biosynthesis were generally up-regulated in S genotype, meanwhile those involved in cell wall degradation (*PE* and *PG*) were down-regulated in S genotype. It could be understand that the DEGs involving in cell walls were inhibited in both genotypes under a high inoculation pressures, however, a more intense defense response in cell wall biosynthesis occurred in S genotype. The induction of PR protein indicates activation of systematic acquired resistance to pathogen, PR protein including PR1, PR2, PR5 and PR10 were identified as positive roles in tomato resistance to *R. solanacearum* (21,24,29)*.* In present study, *PR-2* were detected only in inoculated samples of S genotype and RC1, *PR-5* were down regulated in S genotype meanwhile one *PR-10* showed up regulation in S genotype, their expression were different from those in tomato, indicated a potential different role of them in peanut resistance to *R. solanacearum*.

## Conclusion

In present study, we have investigated the transcriptome in roots of peanut R and S genotypes response to *R. solanacearum* infection at five time points. The dynamics profiles of transcriptome during the infection were roughly characterized. Both genotypes showed a general similarity on transcriptome level at early time points, meanwhile resistance-related DEGs showed distinct genotype-specific expression pattern in response to *R. solanacearum* challenge. The enrichment analyses for the DEGs showed genotype-specific patterns of transcriptome remodelling under *R. solanacearum* challenge. The present work provided the largest unigene dataset for cultivated peanut response to *R. solanacearum* infection, greatly expanded the vision of the genetic basis and improved our understanding on the molecular mechanisms underlying peanut resistance to *R. solanacearum*.

## Methods

### Plant inoculation and tissue harvest

Two peanut genotypes, R genotype J04 and S genotype J62, were provided by Oil Crop Research Institute, Chinese Academy of Agricultural Sciences. Two genotypes greatly differ in resistance to *Ralstonia solanacearum* and are close in genetic background [66]. The seeds were sterilized and germinated on wet double filters paper in a culture dish and transplanted to a porcelain pot full of water when the roots were about 2 cm long, under 16 h photoperiod at 26°C with the relative humidity of 65-80%. The virulent *R. solanacearum* strain (Race 1, biovar 3) was isolated from soil of disease nurseries of Hongan County. Bacterial cells were cultured in CPG medium (pH 7.0) containing 0.1% casamino acids, 1% peptone and 0.5% glucose, at 200 rpm and 28°C in a shaker for 48 hours.

When seedlings were at 3-leaf stage, the root tips were cut to produce wound and then cultivated in a cultivation cabinet. After 24 hours when the root tips were cut, the roots were inoculated with *R. solanacearum* strain race 1 suspension (10^7^cfu/ml) in a culture dishes for 30 min, meanwhile the mock inoculation seedlings were used as control. The roots of seven individual seedlings were sampled at 0, 6, 12, 24, 48 and 72 hours post inoculation. The samples were frozen in liquid nitrogen immediately and stored at -80°C. About 15 individuals of each line were kept for more than 10 days to confirm successful inoculation (Additional file 26: Figure S8).

### Detection of population dynamics of *R. solanacearum*in roots

The roots of infected samples at each time point were vigorously grinded in a mortar for 1 g of roots per 1 ml distilled water, and the serial dilutions of extracted suspensions were made with dilution multiple from 10^3^ to 10^8^_,_ and 0.1 ml aliquots were spread on the surface of a CPG medium then cultured in a CPG medium. The medium is made of 10 g bacto peptone (Difco), 5 g Glucose, 1 g casamino acid (Difco), 15 g bacto agar (Difco), and 1 L distilled water. This medium was supplemented with 1% polymyxin B sulphate (Sigma), 1% crystal violet, 1% bacitracin (Sigma), and 1% cycloheximide (Sigma). After incubating plates at 28°C for 48 hours, colonies of *R. solanacearum* were counted and cfu were calculated per gram of roots. Three replicates were prepared for each sample.

### Construction of the transcriptome

For samples of two genotypes, RNAs were isolated from pooled roots using Trizol reagent (Invitrogen, US), then analysed with Agilent 2100 Bioanalyzer (Agilent Technologies, Santa Clara, CA). The RNA samples were numbered with the sampling time point of 6, 12, 24, 48 and 72 h. For inoculated samples, they were denoted as S6, S12, S24, S48 and S72 for S genotype; and R6, R12, R24, R48 and R72 for R genotype. For control samples, the equal amount of RNA from time points 6, 12 and 24 h were pooled and denoted as RC1 and SC1 for R and S genotype, the pools of RNAs from 48 and 72 h were denoted as RC2 and SC2 for R and for S genotype. Rna-sequencing was carried by Beijing Genomics Institute for Illumina sequencing on a HiSeq2000 system. The raw sequence from fourteen libraries were assembled into comprehensive unigenes using Trinity and TGICL [27, 29].

### Transcriptome functional annotation

The assignment of sequence orientations and functional annotations of the all-unigenes were determined by BLASTx against the followed database, the NCBI nonredundant (Nr) protein database, the Swiss-Prot protein database, the Kyoto Encyclopedia of Genes and Genomes (KEGG) pathway database and the Cluster of Orthologous Groups (COG) database with an E-value cut-off of 1.0e–5. The all-unigenes were assigned GO annotations using Blast2GO (http://www.blast2go.rog/). In addition, unigenes were aligned with the NCBI nucleotide (Nt) databases using BLASTn with an E value of 1.0e–5.

### Expression analysis

First, All reads of each libraries were respectively mapped onto the unigenes using the default parameters by SOAP [67]. Second, the uniquely mapped reads were extracted for quantifing the abundance. Third, Unigene expression was normalized using the value of RPKM (reads per kilobase per million reads). Multiple pairwise comparisons were carried between the data sets of different samples (Additional file 12: Table S2). The absolute value of log2(Ratio) ≥ 1 (under the criterion of P ≤ 0.01 and FDR ≤0.001) were used as threshold to assess the significance of gene expression difference.

### qRT-PCR analysis

The reverse transcripts were performed using an Invitrogen SuperScript Reagent Kit. The primer designed using the Oligo6 software. For RT-PCR, the SYBR^®^ Premix Ex Taq™ (TAKARA) was used on a Bio-Rad CFX96 real-time PCR detection system (Bio-Rad, Hercules, CA). Gene expression was analyzed for samples at 6, 12, 24, 48 and 72 hours post-inoculation of R and S genotypes. All reactions for each gene were performed in triplicate. The relative expression level of each gene among samples was calculated using the 2-△△Ct method with normalization to the internal reference actin gene. The parameters of thermal cycle were 95°C for 30 s, followed by 40 cycles of 95°C for 10 s, 50–56°C for 25 s at a volume of 20 μl.

## Electronic supplementary material

Additional file 1: Figure S1: Length distribution of all unigenes. (TIFF 1 MB)

Additional file 2: Table S1: Summary of annotations of unigenes. (DOCX 13 KB)

Additional file 3: File S1: Result of all unigenes blast against Nr. (ZIP 2 MB)

Additional file 4: File S2: Result of all unigenes blast against Nt. (XLSX 6 MB)

Additional file 5: File S3: Result of all unigenes blast against database of *Arabidopsis*, *Glycine max* and Peanut. (XLSX 8 MB)

Additional file 6: File S4: Result of all unigenes blast against *Ralstonia solanacearum* genome. (XLSX 188 KB)

Additional file 7: File S5: COG annotation of all unigenes. (ZIP 3 MB)

Additional file 8: Figure S2: COG function of all unigenes. (TIFF 2 MB)

Additional file 9: Figure S3: GO classification of unigenes. (TIFF 1 MB)

Additional file 10: File S6: GO category of all unigenes. (ZIP 696 KB)

Additional file 11: File S7: KEGG annotation of all unigenes. (ZIP 3 MB)

Additional file 12: Table S2: The summary of pairwise comparisons. (DOCX 14 KB)

Additional file 13: File S8: All DEGs’ annotations. (XLSX 9 MB)

Additional file 14: Table S3: Differential expressed unigenes of pairwise comparison between data sets. (DOCX 14 KB)

Additional file 15: Table S4: The co-expression pattern of DEGs in R and S data set. (DOCX 20 KB)

Additional file 16: Table S5: The co-expression pattern of DEGs in D data set. (DOCX 17 KB)

Additional file 17: Table S6: The information of DEGs and house-keeping gene used in real-time PCR. (DOC 52 KB)

Additional file 18: Figure S4: The expression validation of DEGs by real-time PCR. (TIFF 1013 KB)

Additional file 19: File S9: DEGs involved in carbohydrate metabolism. (XLSX 27 KB)

Additional file 20: Figure S5: KEGG enrichment analyses of Energy metabolism. (TIFF 988 KB)

Additional file 21: Figure S6: KEGG enrichment analyses of lipid metabolism. (TIFF 2 MB)

Additional file 22: File S10: Defense-related DEGs. (XLSX 135 KB)

Additional file 23: File S11: Phytoalexins biosynthesis-related DEGs. (XLSX 208 KB)

Additional file 24: Figure S7: The expression pattern of cell wall genes. (TIFF 1 MB)

Additional file 25: File S12: DEGs in plant hormone signaling pathway. (XLSX 39 KB)

Additional file 26: Figure S8: The phenotypes of resistant genotype J04 and susceptible genotype J62 after *R. solanacearum* infection. (TIFF 992 KB)
