# Supplementary figures and images for: Dynamics in the resistant and susceptible peanut (Arachis hypogaea L.) root transcriptome on infection with the Ralstonia solanacearum
Source: BMC Genomics. 2014 Dec 7;15(1):1078. doi: 10.1186/1471-2164-15-1078 (PMC4300042; doi:10.1186/1471-2164-15-1078)

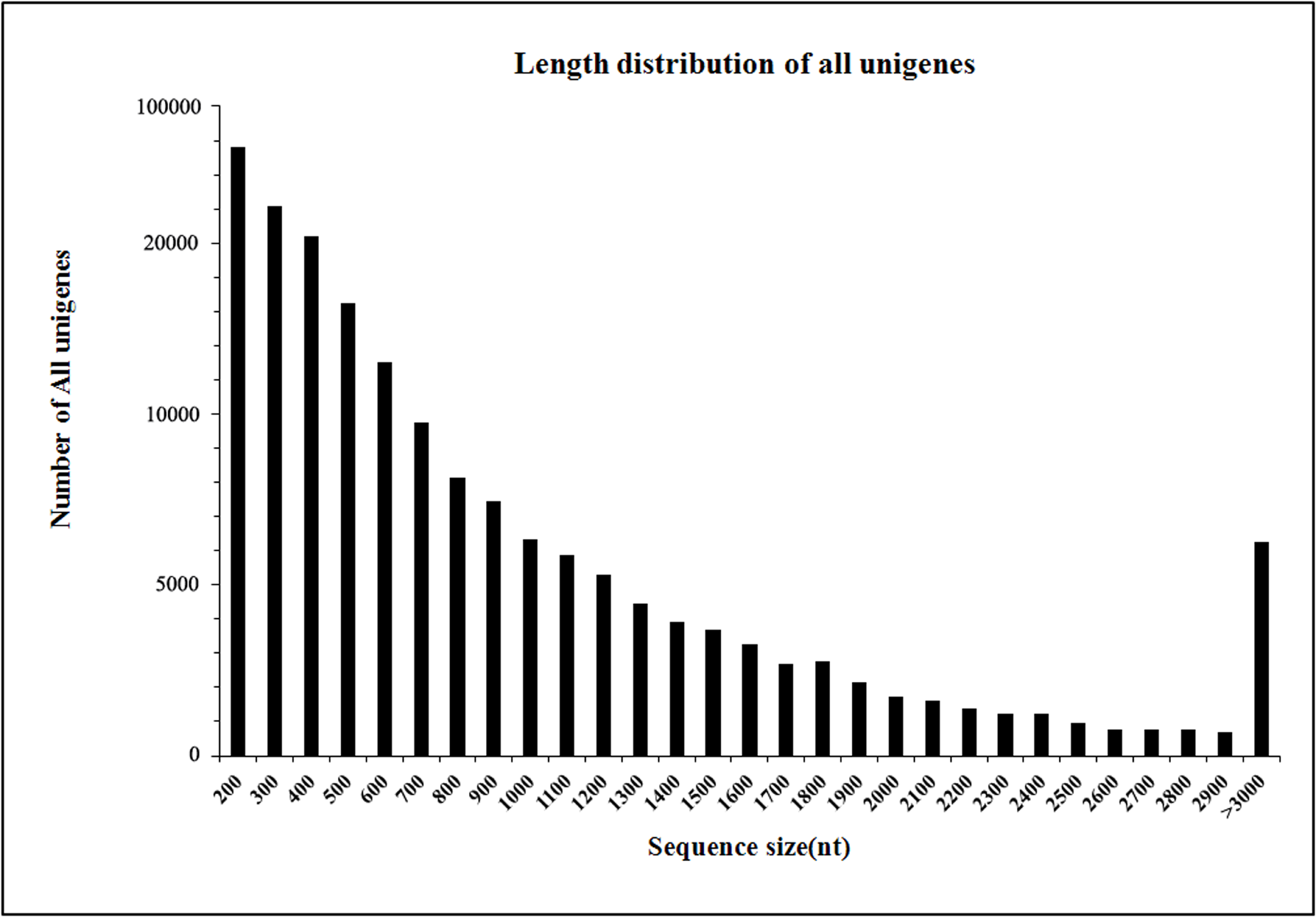

Supplement: Supplementary file 1 — Additional file 1: Figure S1: Length distribution of all unigenes. (TIFF 1 MB) [file 12864_2014_6894_MOESM1_ESM.tiff]

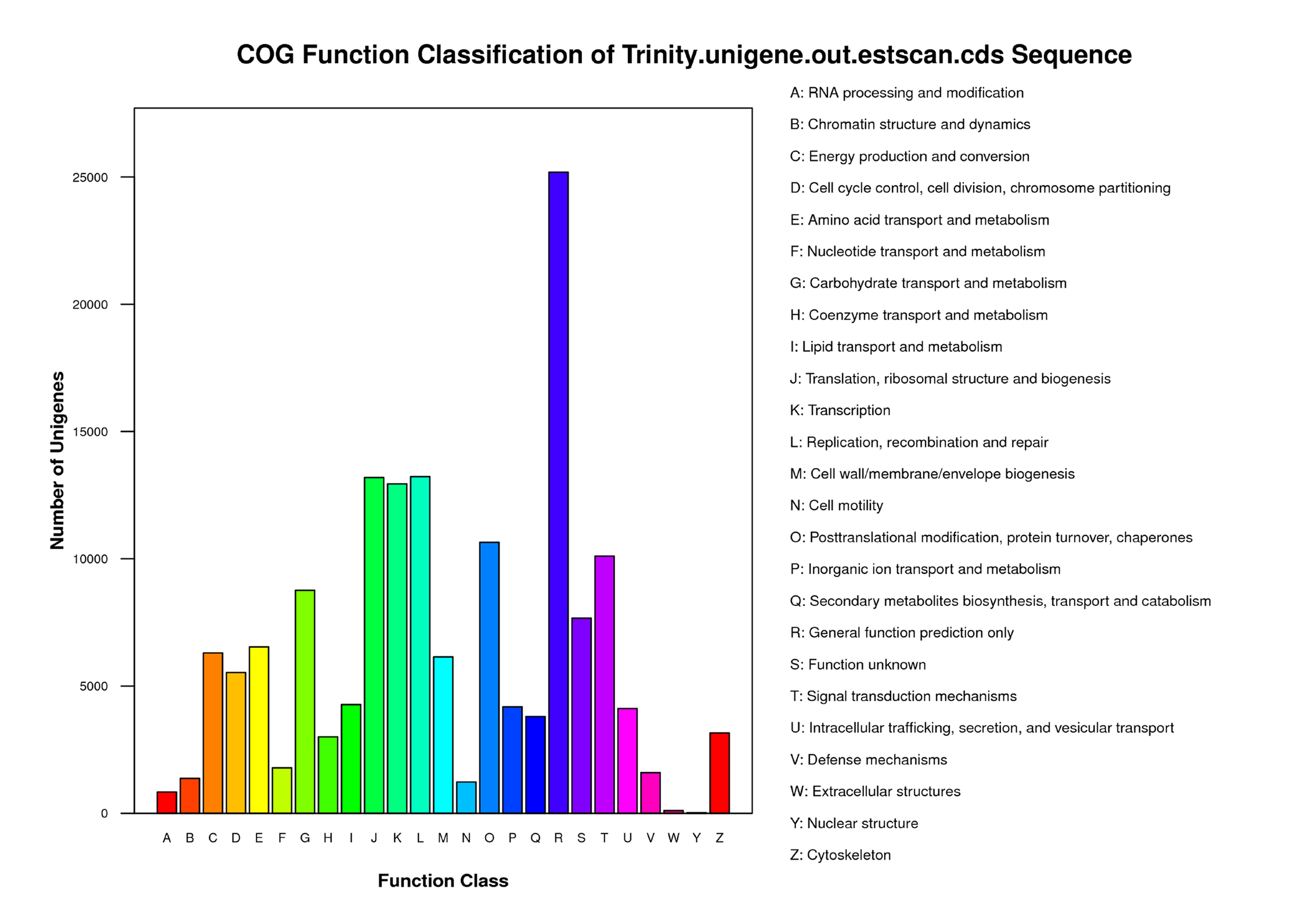

Supplement: Supplementary file 8 — Additional file 8: Figure S2: COG function of all unigenes. (TIFF 2 MB) [file 12864_2014_6894_MOESM8_ESM.tiff]

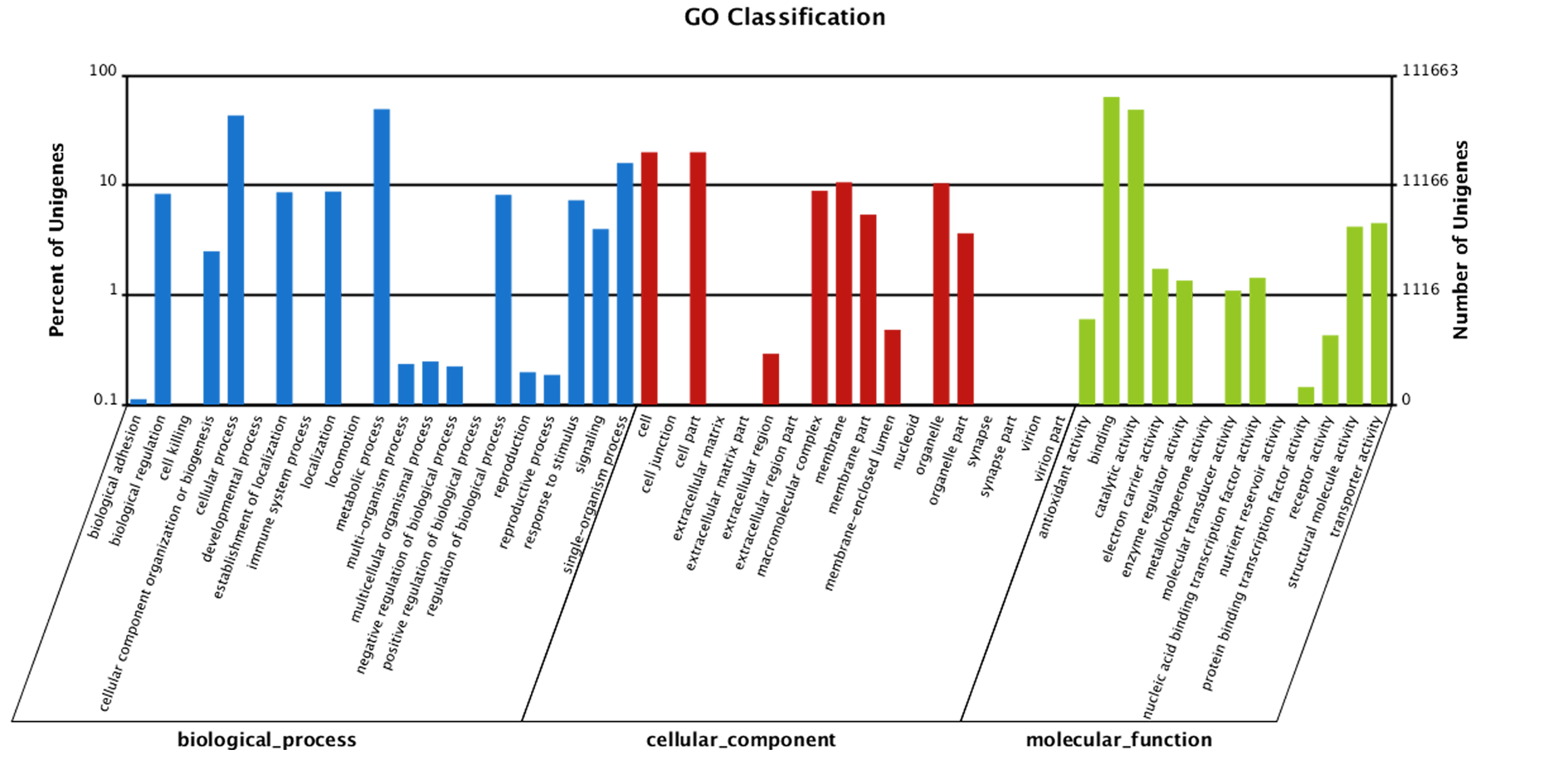

Supplement: Supplementary file 9 — Additional file 9: Figure S3: GO classification of unigenes. (TIFF 1 MB) [file 12864_2014_6894_MOESM9_ESM.tiff]

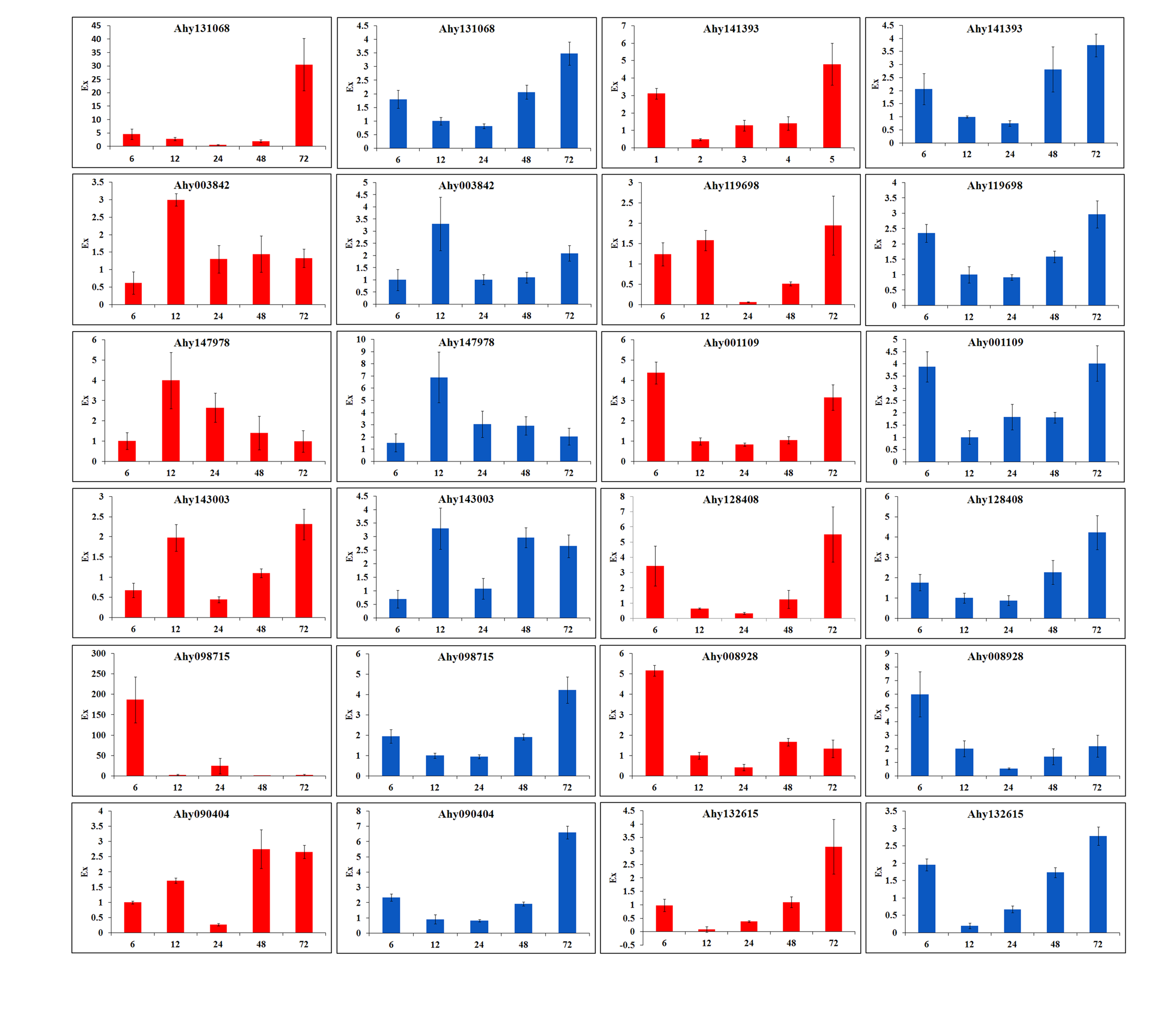

Supplement: Supplementary file 18 — Additional file 18: Figure S4: The expression validation of DEGs by real-time PCR. (TIFF 1013 KB) [file 12864_2014_6894_MOESM18_ESM.tiff]

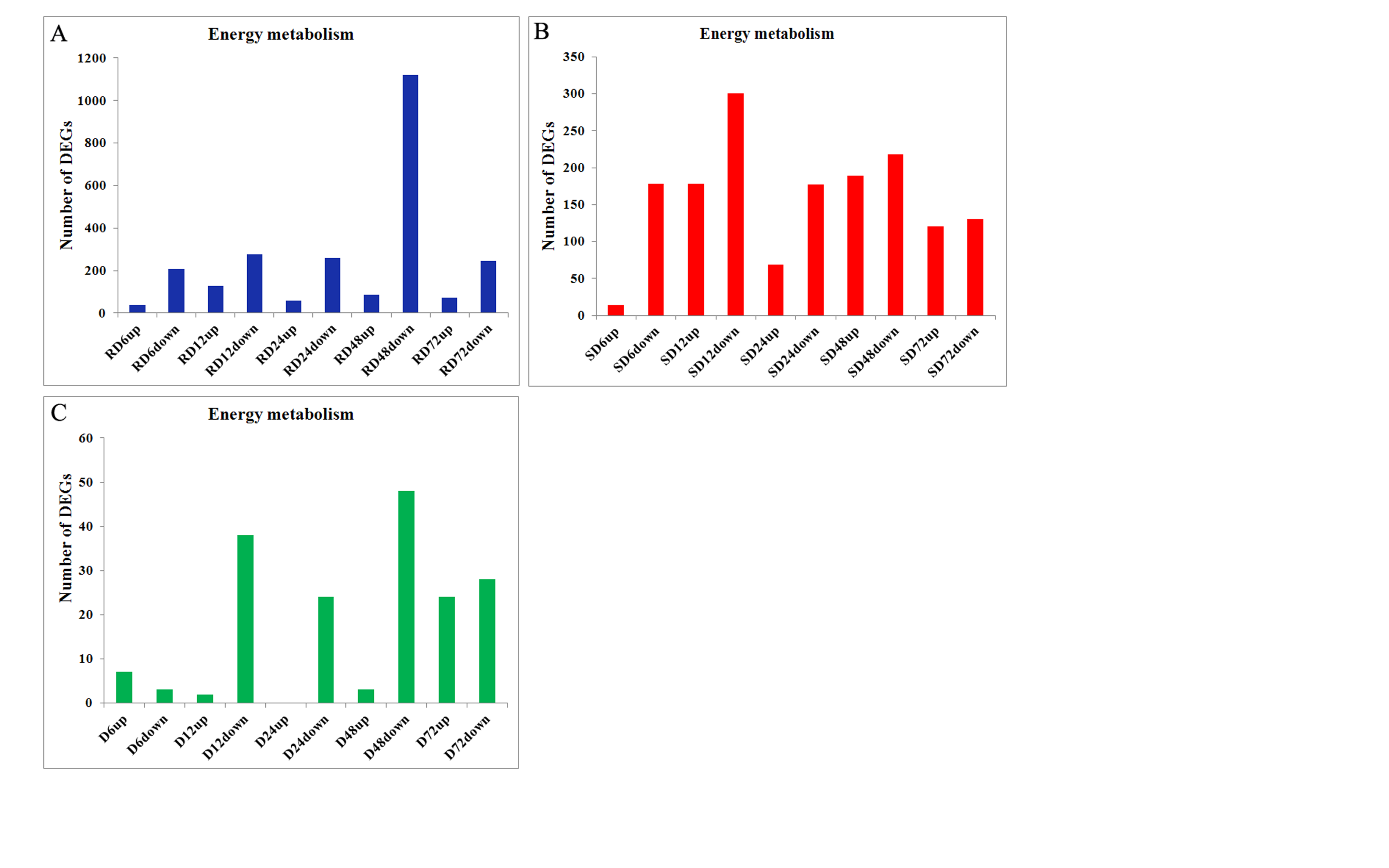

Supplement: Supplementary file 20 — Additional file 20: Figure S5: KEGG enrichment analyses of Energy metabolism. (TIFF 988 KB) [file 12864_2014_6894_MOESM20_ESM.tiff]

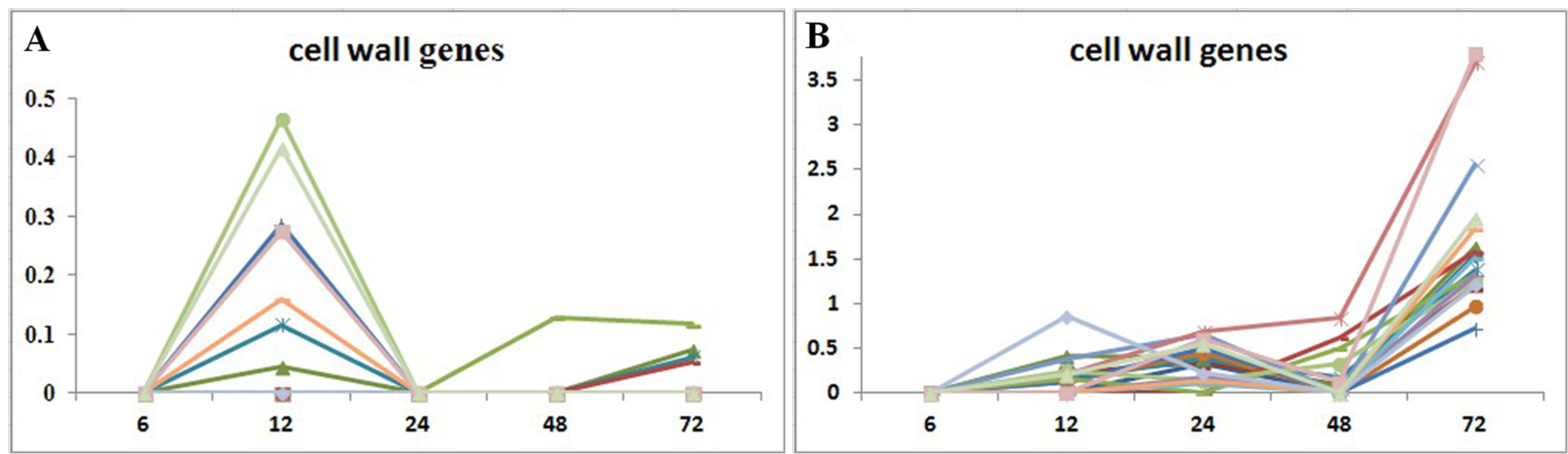

Supplement: Supplementary file 21 — Additional file 21: Figure S6: KEGG enrichment analyses of lipid metabolism. (TIFF 2 MB) [file 12864_2014_6894_MOESM21_ESM.tiff]

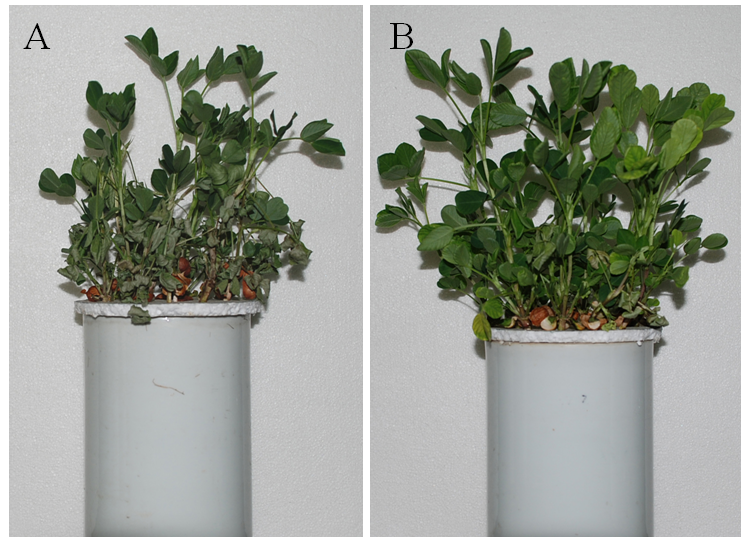

Supplement: Supplementary file 24 — Additional file 24: Figure S7: The expression pattern of cell wall genes. (TIFF 1 MB) [file 12864_2014_6894_MOESM24_ESM.tiff]

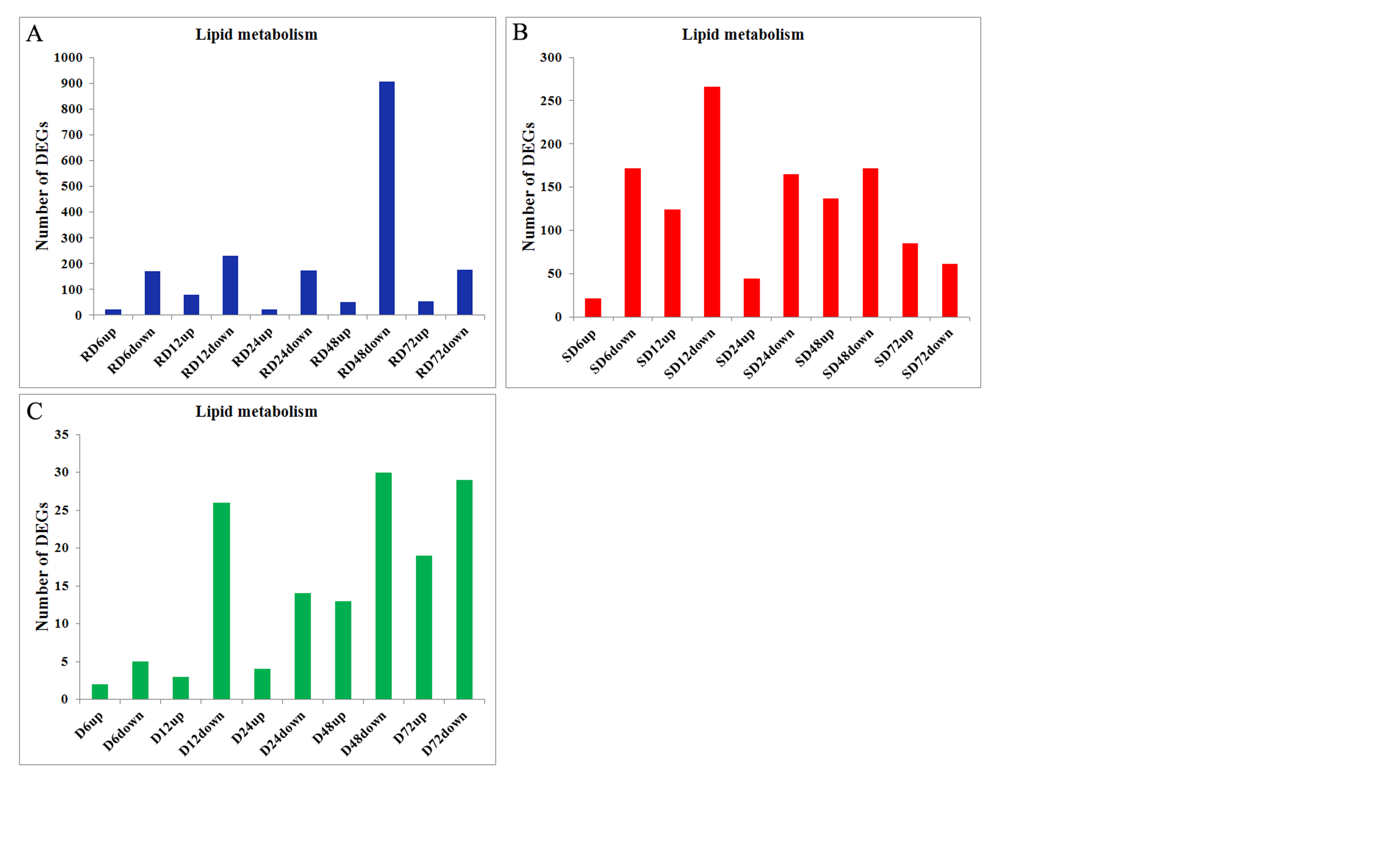

Supplement: Supplementary file 26 — Additional file 26: Figure S8: The phenotypes of resistant genotype J04 and susceptible genotype J62 after R. solanacearum infection. (TIFF 992 KB) [file 12864_2014_6894_MOESM26_ESM.tiff]
