# Supplementary material for: Dynamics in the resistant and susceptible peanut (Arachis hypogaea L.) root transcriptome on infection with the Ralstonia solanacearum
Source: BMC Genomics. 2014 Dec 7;15(1):1078. doi: 10.1186/1471-2164-15-1078 (PMC4300042; doi:10.1186/1471-2164-15-1078)
Supplement: Supplementary file 2 — Additional file 2: Table S1: Summary of annotations of unigenes. (DOCX 13 KB) [file 12864_2014_6894_MOESM2_ESM.docx]

Additional file 2: Table S1. Summary of annotations of unigenes

|  | Sequences(n) | Annotation(n) | Functional classification |
| --- | --- | --- | --- |
| All assembled unigenes | 271 790 |  |  |
| Genes annotations against protein of Nr | 179 641 | 179 641 |  |
| Genes similarity against sequences of Nt | 109 845 | 882 729 |  |
| Genes annotations against Swissport | 117 160 | 789 284 |  |
| Genes annotations against COG | 92 326 | 650 524 | 23 categories |
| Genes annotations against IPRSCAN | 166 580 | 104 3098 | 7839 domains/family |
| Genes annotations against KEGG | 117 185 | 918 004 | 237pathays |
| Go annotations for IPRSCAN hits | 111 663 | 111 663 | 3 main categories 43 sub-categories |
| All annotated unigenes | 179 641 |  |  |
