# Supplementary material for: Dynamics in the resistant and susceptible peanut (Arachis hypogaea L.) root transcriptome on infection with the Ralstonia solanacearum
Source: BMC Genomics. 2014 Dec 7;15(1):1078. doi: 10.1186/1471-2164-15-1078 (PMC4300042; doi:10.1186/1471-2164-15-1078)
Supplement: Supplementary file 12 — Additional file 12: Table S2: The summary of pairwise comparisons. (DOCX 14 KB) [file 12864_2014_6894_MOESM12_ESM.docx]

| Comparision | Resulted Data set | Comparision | Resulted Data set |
| --- | --- | --- | --- |
| R6 vs RC1 | RD6 | S6 vs SC1 | SD6 |
| R12 vsRC1 | RD12 | S12 vsSC1 | SD12 |
| R24 vsRC1 | RD24 | S24 vsSC1 | SD24 |
| R48 vs RC2 | RD48 | S48 vs SC2 | SD48 |
| R72 vs RC2 | RD72 | S72 vs SC2 | SD72 |
| R6 vs S6 | D6 | R12 vsS12 | D12 |
| R24 vsS24 | D24 | R48 vsS48 | D48 |
| R72 vs S72 | D48 |  |  |

Additional table 2. The pairwise comparisons between inoculated and control samples for respective R and S genotypes, and pairwise comparisons between inoculated samples from R and S genotyps.
