# Supplementary material for: Dynamics in the resistant and susceptible peanut (Arachis hypogaea L.) root transcriptome on infection with the Ralstonia solanacearum
Source: BMC Genomics. 2014 Dec 7;15(1):1078. doi: 10.1186/1471-2164-15-1078 (PMC4300042; doi:10.1186/1471-2164-15-1078)
Supplement: Supplementary file 14 — Additional file 14: Table S3: Differential expressed unigenes of pairwise comparison between data sets. (DOCX 14 KB) [file 12864_2014_6894_MOESM14_ESM.docx]

Additional Table 3. Differential expressed unigenes of pairwise comparison between data sets.

| Comparision | Up | Down | Comparision | Up | Down |
| --- | --- | --- | --- | --- | --- |
| R6 vs RC1 | 1375 | 7213 | S6 vs SC1 | 636 | 6974 |
| R12 vsRC1 | 6528 | 9019 | S12 vsSC1 | 10159 | 9916 |
| R24 vsRC1 | 1163 | 8818 | S24 vsSC1 | 2340 | 6895 |
| R48 vs RC2 | 2779 | 12942 | S48 vs SC2 | 8581 | 6451 |
| R72 vs RC2 | 2953 | 12683 | S72 vs SC2 | 5477 | 3234 |
| R6 vs S6 | 981 | 552 | R12 vsS12 | 1338 | 4714 |
| R24 vsS24 | 595 | 3651 | R48 vsS48 | 1191 | 5901 |
| R72 vs S72 | 2610 | 5772 |  |  |  |

Up, the numbers of DEGs up-regulated in inoculated samples; Down, the the numbers of DEGs up-regulated in inoculated samples.
