# Supplementary material for: Dynamics in the resistant and susceptible peanut (Arachis hypogaea L.) root transcriptome on infection with the Ralstonia solanacearum
Source: BMC Genomics. 2014 Dec 7;15(1):1078. doi: 10.1186/1471-2164-15-1078 (PMC4300042; doi:10.1186/1471-2164-15-1078)
Supplement: Supplementary file 15 — Additional file 15: Table S4: The co-expression pattern of DEGs in R and S data set. (DOCX 20 KB) [file 12864_2014_6894_MOESM15_ESM.docx]

Additional Table 4. The co-expression pattern of DEGs in R and S data set.

| 6 | 12 | 24 | 48 | 72 | R | S | R and S |
| --- | --- | --- | --- | --- | --- | --- | --- |
| 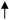 | 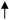 | 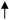 | 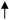 | 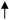 | 11 | 6 | 1 |
| 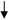 | 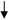 | 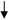 | 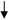 | 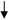 | 1336 | 648 | 253 |
| 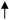 | 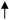 | 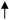 | 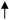 |  | 28 | 108 | 7 |
| 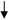 | 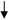 | 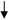 | 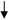 |  | 71 | 268 | 16 |
| 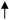 | 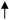 | 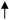 |  |  | 13 | 12 | 0 |
| 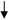 | 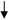 | 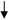 |  |  | 1979 | 1911 | 851 |
|  |  |  | 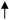 | 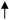 | 584 | 684 | 0 |
|  |  |  | 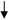 | 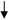 | 8107 | 988 | 203 |
| 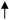 |  |  | 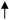 | 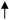 | 318 | 1 | 0 |
| 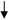 |  |  | 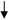 | 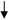 | 68 | 8 | 1 |
|  |  | 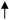 |  | 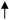 | 86 | 592 | 0 |
|  | 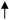 | 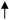 | 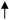 | 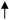 | 249 | 172 | 0 |
|  | 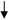 | 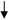 | 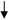 | 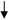 | 209 | 72 | 33 |
|  | 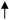 | 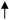 | 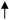 |  | 75 | 95 | 18 |
|  | 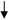 | 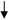 | 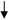 |  | 156 | 395 | 37 |
|  | 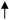 | 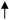 |  |  | 218 | 4 | 4 |
|  | 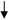 | 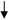 |  |  | 551 | 155 | 28 |
| 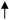 | 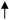 |  |  |  | 74 | 3 | 3 |
| 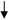 | 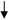 |  |  |  | 112 | 244 | 20 |
| 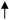 |  | 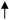 |  |  | 13 | 0 | 0 |
| 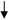 |  | 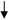 |  |  | 1608 | 1899 | 632 |
|  | 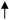 |  | 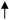 | 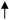 | 112 | 142 | 4 |
|  | 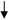 |  | 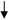 | 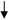 | 5 | 4 | 0 |

The up arrow indicates the up-regulation of DEGs, the down arrow indicates the down-regulation of DEGs. R, DEGS in resistant genotype; S, DEGs in susceptible genotype; R and S, DEGs shared by R and S genotypes.
