# Supplementary material for: Dynamics in the resistant and susceptible peanut (Arachis hypogaea L.) root transcriptome on infection with the Ralstonia solanacearum
Source: BMC Genomics. 2014 Dec 7;15(1):1078. doi: 10.1186/1471-2164-15-1078 (PMC4300042; doi:10.1186/1471-2164-15-1078)
Supplement: Supplementary file 16 — Additional file 16: Table S5: The co-expression pattern of DEGs in D data set. (DOCX 17 KB) [file 12864_2014_6894_MOESM16_ESM.docx]

Additional Table 5. The co-expression pattern of DEGs in D data set.

| Time points | | | | |  |
| --- | --- | --- | --- | --- | --- |
| 6 | 12 | 24 | 48 | 72 | No |
|  |  |  |  |  | 57 |
|  |  |  |  |  | 47 |
|  |  |  |  |  | 11 |
|  |  |  |  |  | 7 |
|  |  |  |  |  | 8 |
|  |  |  |  |  | 9 |
|  |  |  |  |  | 1 |
|  |  |  |  |  | 17 |
|  |  |  |  |  | 30 |
|  |  |  |  |  | 9 |
|  |  |  |  |  | 119 |
|  |  |  |  |  | 7 |
|  |  |  |  |  | 12 |
|  |  |  |  |  | 19 |
|  |  |  |  |  | 156 |
|  |  |  |  |  | 2 |
|  |  |  |  |  | 114 |
|  |  |  |  |  | 22 |
|  |  |  |  |  | 46 |
|  |  |  |  |  | 29 |
|  |  |  |  |  | 151 |
|  |  |  |  |  | 15 |
|  |  |  |  |  | 285 |
|  |  |  |  |  | 6 |
|  |  |  |  |  |  |
