# Supplementary material for: Dynamics in the resistant and susceptible peanut (Arachis hypogaea L.) root transcriptome on infection with the Ralstonia solanacearum
Source: BMC Genomics. 2014 Dec 7;15(1):1078. doi: 10.1186/1471-2164-15-1078 (PMC4300042; doi:10.1186/1471-2164-15-1078)
Supplement: Supplementary file 17 — Additional file 17: Table S6: The information of DEGs and house-keeping gene used in real-time PCR. (DOC 52 KB) [file 12864_2014_6894_MOESM17_ESM.doc]

Additional Table 6. The information of DEGs and house-keeping gene used in real-time PCR.

| Gene | Annotation | Homology | E-value | Primer sequence |
| --- | --- | --- | --- | --- |
| Ahy141393 | Histidine kinase | XP_003524900.1 | 3E-72 | F: 5' GTT GAC GAA GTG CGA CGA AAT 3'  R: 5' CCC AGA CCC ATT CGA GCT A 3' |
| Ahy003842 | Leucine-rich repeat/extensin | XP_003526272.1 | 0 | F: 5' GCC CAA TTC CTT ATA GCC T 3'  R: 5' TGC GGC GAT CAT CA 3' |
| Ahy119698 | ERF5 | NP_001241150.1 | 2E-92 | F: 5' AAT GAT GAA GAG GGT AGT GAA 3'  R: 5' AGG AGG AAC GCT AAA GAT 3' |
| Ahy147978 | NBS-LRR | XP_003553392.1 | 2E-80 | F: 5' GGT CTT CGC AAA GTG CTA C3'  R: 5' CAG GGC AAT CTT CGA T 3' |
| Ahy001109 | NB-LRR | XP_003522023.1 | 4E-95 | F: 5' GTT GTT GGC AAG CAC GAA G 3'  R: 5' GCG GTT TCA AGT TAT CGA G 3' |
| Ahy143003 | Respiratory burst oxidase | XP_003533800.1 | 0 | F: 5' TTG CAT TTG CCA TAG C 3'  R: 5' TGA TGA GTT GCG AGT GTA A3' |
| Ahy128408 | Ethylene-production | XP_003541491.1 | 9E-22 | F: 5' GCA GCA GAT GGT TAG CAA A 3'  R: 5' TGG GCA GCA TTA GG 3' |
| Ahy098715 | Calreticulin | Q40401.1 | 1E-44 | F: 5' TGA AGA TTG GGA CGA CGA A 3'  R: 5' GCT TGT AGT CAG GGT TGG C 3' |
| Ahy008928 | Serine/threonine-protein kinase | XP_003525044.1 | 9E-102 | F: 5' GGA AAT ATG CCA TAG GGA TCT T 3'  R: 5' TTC CGG TGC AAT ATA AGC3' |
| Ahy090404 | Endoglucanase | XP_004171003.1 | 4E-33 | F: 5' CGG ACA AGT TCG ATG GC 3'  R: 5' TTT TGT CAA TAA CGG TGG C 3' |
| Ahy132615 | Predicted protein | XP_002332644.1 | 1E-69 | F: 5' AAC GTA GGA TAT TTG GGT TGA 3'  R: 5' AAT TGG TGG ATG AAT GGT TGA A3' |
| Ahy147979 | RGA2 | XP_003553392.1 | 2E-76 | F: 5' ACA TTT GAG ACT CTA CCT CGT T 3'  R: 5' TTG AAG CTC CAC ATA GCC A 3' |
| Actin |  | EG029307 |  | F: 5' TAA GAA CAA TGT TGC CAT ACA GA 3'  R: 5' GTT GCC TTG GAT TAT GAG C 3' |
|  |  |  |  |  |
